# The cortical scene processing network emerges in infancy, prior to independent navigation experience

**DOI:** 10.1073/pnas.2513126123

**Published:** 2026-07-28

**Authors:** Frederik S. Kamps, Emily M. Chen, Haoyu Du, Heather L. Kosakowski, Ariel Fuchs, Nancy Kanwisher, Rebecca Saxe

**Affiliations:** ^a^ Department of Psychology, University of Edinburgh, Edinburgh EH8 9JZ, United Kingdom; ^b^ Department of Brain and Cognitive Sciences, Massachusetts Institute of Technology, Cambridge, MA 02139; ^c^ Department of Psychology, Stanford University, Stanford, CA 94305; ^d^ Department of Psychology, University of California San Diego, La Jolla, CA 92093; ^e^ Department of Psychology, University of Southern California, Los Angeles, CA 90089

**Keywords:** development, category selectivity, scene perception, PPA, fMRI

## Abstract

Sighted people rely on vision to recognize and navigate the local environment. By adulthood, human cortex contains at least three regions that respond selectively to visual scene information, but it remains unknown when or how these regions develop. One hypothesis is that development of scene selectivity depends on passive exposure to the low-level visual statistics of scenes. Another hypothesis is that development depends on active experience using scene information to plan and guide navigation. Using ecological momentary assessment, we measured the quantity of forward-facing ego-motion and active navigation that infants experienced in the first months of life. By age 5 mo, infants cumulatively experienced around 340 h of forward facing ego-motion, almost exclusively passive. Next we used functional MRI in infants aged 2 to 9 mo to measure neural responses to scenes. Awake infants watched videos of ego-motion through real-world scenes, as well as videos of faces, objects, and scrambled videos. We found stronger responses to scenes than control conditions in the location of all three cortical scene regions. Responses in infant scene regions a) were dissociable from those in nearby face and object regions; b) could not be fully explained by low-level visual features; and c) were found even in subsamples of infants with no locomotor experience. Thus, the basic signature of scene selectivity emerges prior to independent navigation and with limited passive visual exposure to the diagnostic visual statistics of scenes.

## Significance

Despite extensive work on the functional organization of scene processing in the human adult visual cortex, little is known about how these responses initially develop. Here, we used ecological momentary assessment and functional MRI in awake human infants aged 2 to 9 mo. All three regions of the known visual scene processing network were scene selective, even in infants with limited visual exposure and no active experience using visual scene information to plan and guide independent navigation (e.g., by crawling). These findings constrain theories of how scene selectivity develops in high-level visual cortex.

Sighted adults adeptly use vision to recognize and find their way through the local environment or “scene.” The foundations of this ability develop early in infancy and toddlerhood. For example, 18-mo-olds use the geometry of the visual environment (e.g., the layout of walls) to navigate to a previously learned location (1), while 8-mo-olds visually distinguish safe vs. unsafe navigational affordances (e.g., steep vs. shallow visual cliffs) (2). By adulthood, the human brain contains at least three regions that respond selectively to visual scenes, and which play a causal role in scene processing and navigation (3): the occipital (OPA) (4), parahippocampal (PPA) (5), and medial place areas (MPA; also known as the retrosplenial complex) (6, 7).

How does cortical scene selectivity develop? One theoretical framework emphasizes the role of passive visual exposure to low-level visual features (e.g., refs. 8–10). On this account, regions that will later develop scene selectivity are initially driven by peripheral visual input and/or low-level visual features that covary with such input, including high spatial frequency content and rectilinear features. Passive exposure to the visual statistics of scenes leads to the frequent coactivation of these features across development, eventually driving the development of higher-level scene selectivity (i.e., responses to scenes over and above a simple combination of this initial set of low-level features). We will refer to this as the “passive exposure” framework. Given that all waking life occurs embedded in a scene, one prediction of the passive exposure framework is that scene responses will increase gradually with age.

A second framework suggests that infants are born not only with basic sensorimotor functions but also with endogenous motivation to learn about the spatial environment and how to navigate through it (e.g., refs. 11–13). On this view, development of higher-level scene selectivity depends on active experience using visual scene information in the service of functional goals, as when planning and guiding navigation. For example, dark-reared kittens develop greater visual cliff sensitivity when they gain active navigation experience (compared to yoked passive exposure to the same space in a “kitten carousel”) (14). Similarly, human infants’ visual cliff sensitivity is related to their locomotor status and ability (15), and prereaching infants given active reaching experience (with “sticky mittens”) show greater attention to goal-directed actions than infants without active experience (16). We will refer to this as the “active experience” framework. Infants are born incapable of independent navigation, and only gain their first experience actively planning and guiding their own navigation late in infancy (i.e., by learning to locomote). One prediction of the active experience framework then is that scene-selective responses emerge only at or after locomotor onset, when scene information becomes relevant for navigation planning.

Critically, while the frameworks above provide a roadmap for theorizing about how cortical scene selectivity develops in humans, they are relatively unconstrained by current empirical evidence. Although numerous studies have demonstrated scene selectivity in children 5 y and older (17–22; see also ref. 23), there are far fewer measurements of these cortical regions in younger children and infants. To address that gap, our lab pioneered methods using fMRI and fNIRS to study scene regions in awake infants, and found that the basic preference for scenes (relative to faces) is already established by at least 6 mo (24, 25), with at least one region, the PPA, showing a selective response to scenes relative to a number of control conditions (i.e., objects, faces, and bodies) (26). Similarly, recent EEG studies have found that dissociable responses to scenes vs. other categories are detectable as early as 6 to 8 mo (27, 28). Despite these advances, however, it is still unclear when selectivity first develops throughout the network and what role active experience and passive exposure play in shaping the emergence of cortical scene selectivity.

To fill these gaps, we scanned awake 2 to 9-mo-old infants while viewing videos of scenes and control videos. To ensure quality fMRI data collection, we used methods similar to Kosakowski et al. (26), including a custom infant head coil for improving temporal signal to noise ratio (tSNR) and strict head motion thresholds for data inclusion. To provide the most powerful test of scene-selectivity we could devise, we first conducted a pilot fMRI experiment in adults, in which we measured responses to 172 candidate scene videos and selected only the top 60 stimuli eliciting the strongest responses in adult scene regions for the current experiment (see *SI Appendix*, Fig. S1 for further details on stimulus selection). Our preregistered predictions were tested using this first dataset (N = 21 sessions from 17 infants; the “preregistered sample”). Then, to maximize statistical power, we combined these data with infants from a prior sample (26). We also report exploratory analyses from this combined dataset (N = 36 sessions from 32 infants; the “full sample”). Additionally, because little is known about infants’ early visual experience of scenes, we conducted an ecological momentary assessment (EMA) study to measure the time course and quantity of infants’ passive exposure to scenes (including the characteristic optic flow created by forward-facing motion) in the months prior to navigation experience.

Measuring responses in infants’ cortex allowed us to preregister and test several hypotheses about the origin of scene-selective responses and the roles of passive exposure and active experience in their development (minor deviations from preregistered analysis plans are noted throughout the text). First, we test when stronger responses to scenes than all control stimuli (i.e., scene selectivity) first appear, and whether these responses are localized to the anatomical vicinity of scene regions in adults. Second, if development of scene selectivity depends on active navigation experience, then scene-selective responses will be detectable only in infants capable of independent locomotion. Our sample contained mostly precrawling infants, allowing us to test the alternative hypothesis that scene selectivity develops prior to locomotion. Third, if development of scene selectivity is learned from passive exposure to visual scene statistics, then the earliest scene responses will be explained by low-level visual features (especially in younger infants), and scene selectivity will gradually increase with age (a proxy for passive exposure to visual scene statistics).

## Results

### Estimating Scene Experience in Infancy.

**Fig. 1.**
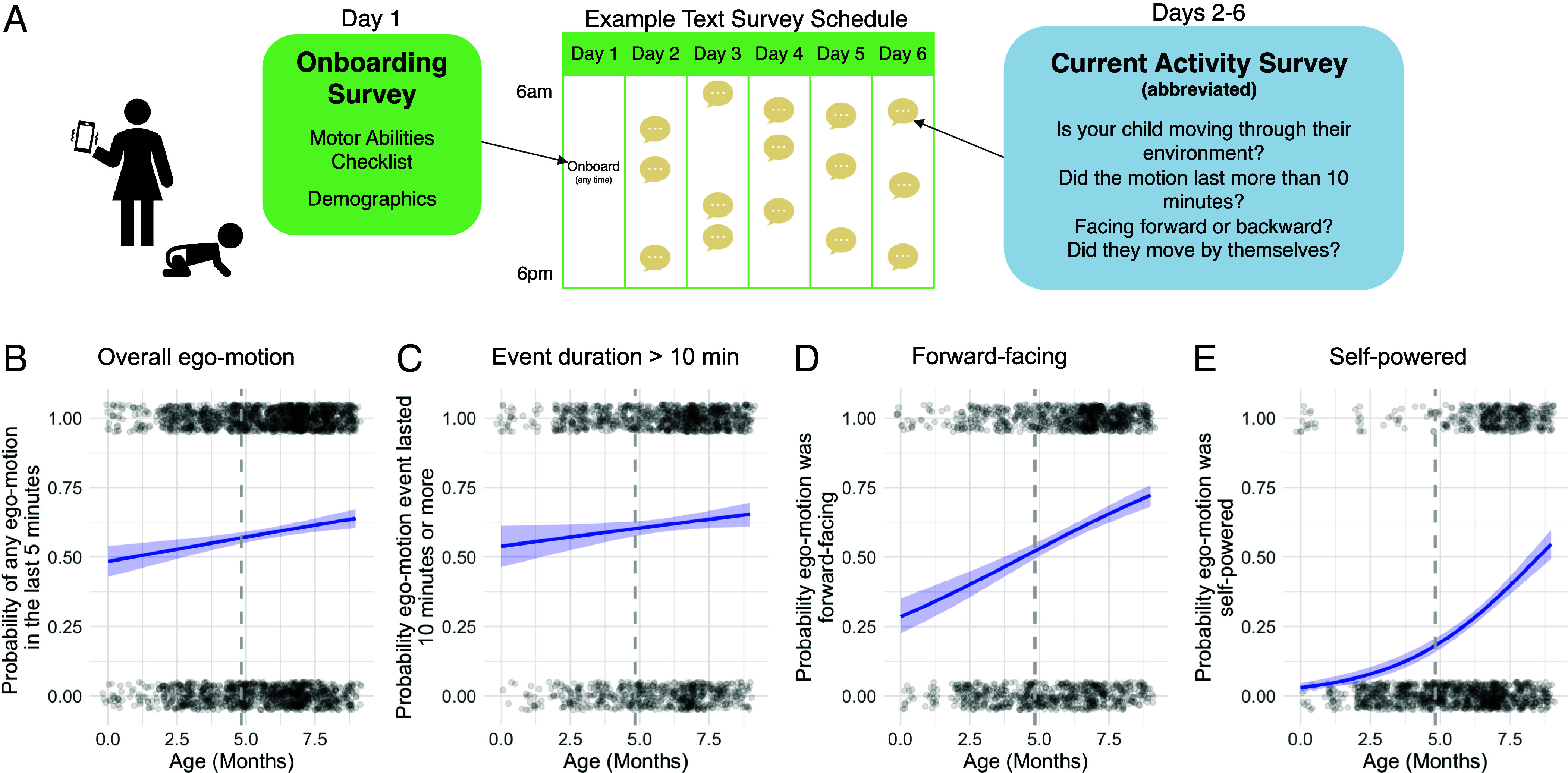
EMA study of experience with ego-motion through the environment across the first 9 mo. (*A*) Schematic describing study design. On Day 1, parents registered for the study online, and completed an initial onboarding survey including a motor abilities checklist and basic demographic questions. On Days 2 to 6, parents received 3 text messages at random times between 6 am and 6 pm. Each text included a link to a short survey about their child’s current experiences with ego-motion through the environment (the unabbreviated survey is provided in *SI Appendix*, *Supplemental Materials*). (*B*) Proportion of survey responses indicating that yes their child had experienced any kind of ego-motion in the past 5 min, as a function of age. (*C*) Proportion of the “yes” ego-motion responses presented in (*B*) where the parent also indicated the ego-motion event lasted 10 min or more, as a function of age. (*D*) Proportion of yes ego-motion responses where parents indicated the child was facing forward during the ego-motion, as a function of age. (*E*) Proportion of yes ego-motion responses where parents indicated the motion was self-powered (rather than generated by another person or object), as a function of age. For all plots, blue lines indicate the fit of a logistic regression model with shaded CI. Dots jittered around 0 and 1 indicate individual survey responses. Gray vertical lines indicate the youngest age at which any parent indicated their child was able to crawl. Sample includes 222 caregivers with 2,657 total responses.

Before we tested how passive and active visual experiences are related to the emergence of scene selectivity, we quantified infants’ visual experience with scenes over the first 9 mo of life, before and after the onset of independent locomotion (crawling and walking). Surprisingly little is known about infant’s visual experience with forward-facing ego-motion through the environment (e.g., refs. 29–33), a key source of visual experience with scenes. To quantify infants’ early scene experiences, we conducted an EMA study in which N = 222 parents or caregivers of infants up to age 9 mo old (mean = 5.7 mo) responded to 15 texts across 5 d, sent at random times between 6 am and 6 pm (Fig. 1*A*). With each text, parents reported whether their child i) was awake, ii) had been in motion within the last 5 min, and if so, whether the motion was iii) self- or other-powered, iv) forward- or backward-facing, and v) relatively brief (i.e., 5 min or less) or extended in duration (i.e., 10 min or more). On the first day of the study, we also asked about infants’ current locomotor status and related motor abilities.

Results are shown in Fig. 1 *B*–*E*. Overall ego-motion increased steadily with age, and abundant experience was reported prior to locomotor onset (Fig. 1*B*). Duration of ego-motion bouts followed a similar progression (Fig. 1*C*). Forward-facing motion was present and increasing prior to the transition to self-powered motion (Fig. 1*D*). As expected, ego-motion was initially other-powered and abruptly shifted toward self-powered motion with crawling onset (Fig. 1*E*). Infants already faced forward from the first few months, with forward-facing motion increasing sharply with age. Similar results were found when accounting for children’s wakefulness at the time of response.

These data provide empirical support for several assumptions underlying our predictions for the brain imaging data: i) experience with forward-facing ego-motion begins early, prior to crawling, and accumulates steadily with age, suggesting age is a reasonable proxy for exposure to the relevant visual statistics across the first months of life; ii) human infants have little-to-no experience with active navigation (via self-powered motion) prior to 5 mo of age; and iii) forward-facing ego-motion through scenes occurs before and independently of active locomotion, so effects of active locomotor experience are potentially separable from those of passive visual exposure to optic flow patterns characteristic of scenes.

We also used the EMA data to estimate infants’ total (lifetime) hours of ego-motion experience (details of calculations are provided in the *SI Appendix*, *Supplemental Materials*). We estimate that infants ages 2 (to 9) mo old (the age range of our infant fMRI sample) have experienced approximately 146 h (to 1,427 h for 9 mo olds) of awake ego-motion, and 62 (to 1,223) hours of forward-facing ego-motion (i.e., as depicted in our scene stimuli). A 4.9 mo old infant (the median age of the full fMRI sample) is estimated to have experienced 522 h of ego-motion, and 338 h of forward facing ego-motion, almost all of it passive.

### The Cortical Scene Network Is Scene Selective in Infancy.

**Fig. 2.**
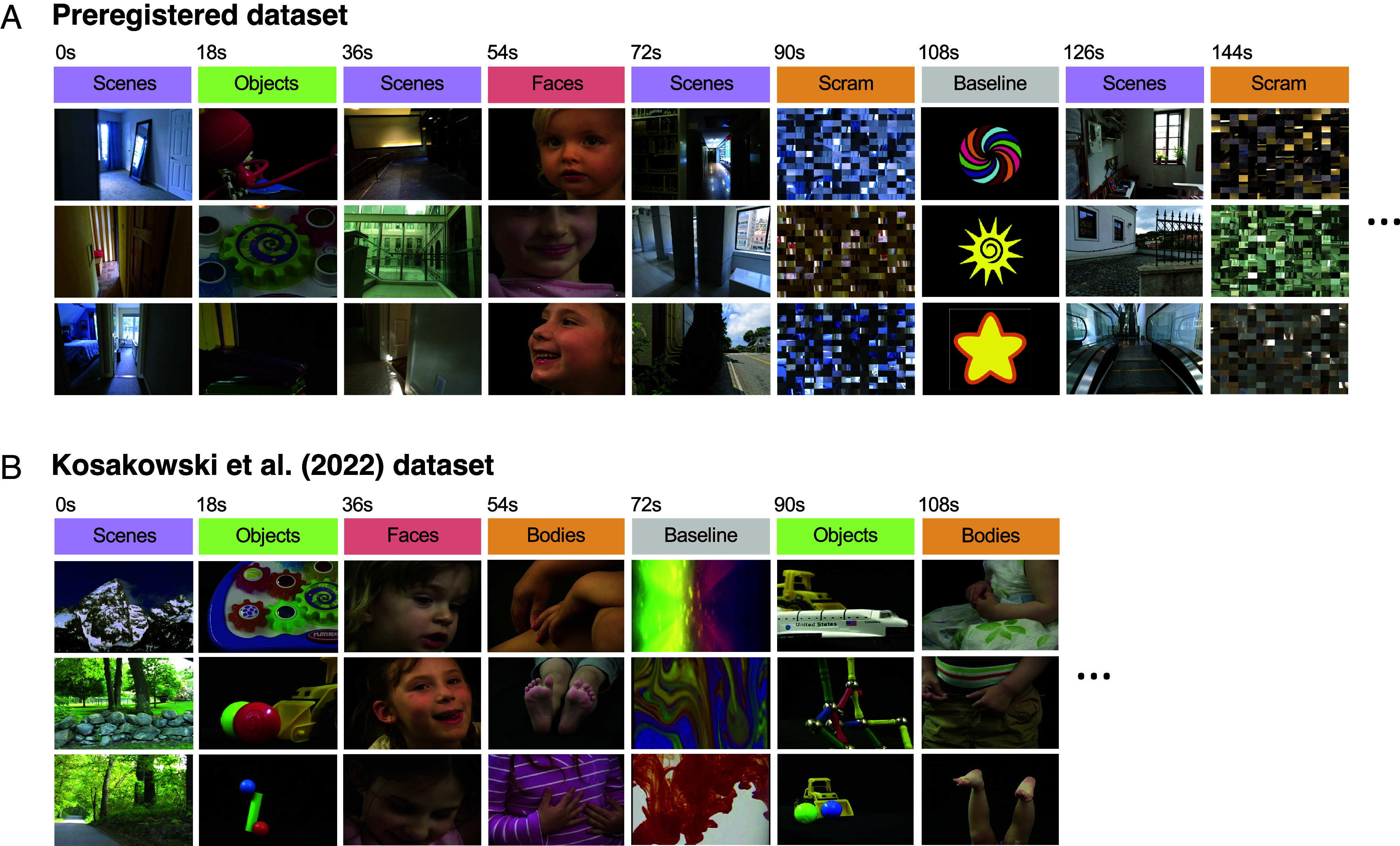
fMRI experimental design and example frames from video stimuli. (*A*) An example block sequence from the preregistered dataset, with example frames from the movie stimuli shown below each 18 s block. Scene blocks were shown every other block, resulting in three times as many scene blocks as object, face, or scrambled scene blocks. Baseline stimuli included colorful, attention-getter object stimuli. (*B*) An example block sequence for data from Kosakowski et al. (26), with example frames from the movie stimuli shown below each 18 s block. Each condition was presented the same number of times. Baseline stimuli included swirling, abstract patterns.

Having established that effects of passive exposure and active experience are potentially dissociable, we next tested whether 2 to 9-mo-old infants show scene responses in similar regions to adults. Infants in the preregistered sample watched blocks of videos depicting scenes, objects, faces, and block-scrambled versions of the scene stimuli, along with baseline stimuli consisting of colorful spinning objects (Fig. 2). Infants from Kosakowski et al. (26) saw similar stimulus categories (scenes, objects, faces, and bodies; baseline stimuli consisted of abstract, swirling colors).

**Fig. 3.**
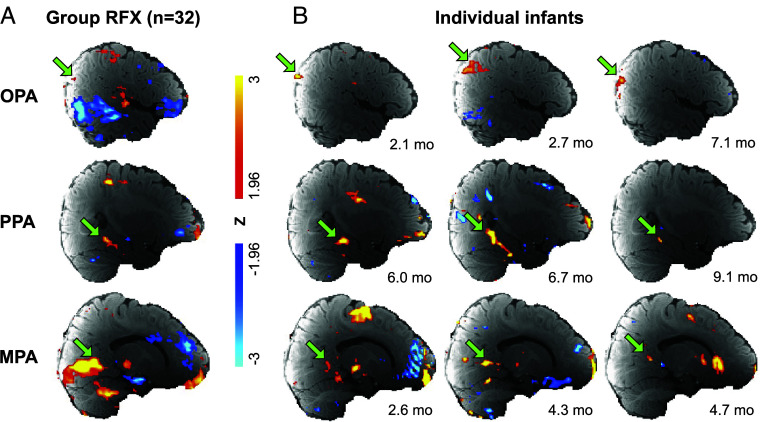
Whole-brain activations to scenes. (*A*) Group random effects analysis for the contrast of scenes (warm) vs. the average of all control conditions (cool) in OPA (*Top*), PPA (*Middle*), and MPA (*Bottom*). For the group random effects analysis, activations in scene regions were not significant after accounting for multiple comparisons. (*B*) Whole brain maps from individual infants showing scene responses (warm) compared to the average of all control conditions (cool) for OPA (*Top*), PPA (*Middle*), and MPA (*Bottom*). Data are shown at a low, uncorrected threshold (*P* < 0.01; transformed to z for visualization). Green arrows indicate scene activations in the regions of interest. All contrast maps are overlaid on a representative anatomical image of an infant brain.

We conducted initial exploratory analyses of voxel-wise responses across the whole brain, testing for greater response to scenes than the average of all control conditions. For each potential infant subject, we defined “subruns” as portions of the originally acquired data during which infants were awake and still (see *Methods* for full details on subrun definition). The preregistered dataset included an average of 524.9 vol. (26.3 min) per infant (range: 199 to 1,542), with an average of 71.2% (range: 39.3 to 92.7%) of volumes below the motion threshold of 0.5 mm or degrees. The Kosakowski et al. (26) dataset included an average of 533.9 vol. (26.7 min; range: 207 to 1,182 vol.) per infant, with an average of 61.5% (range: 32.8 to 86.1%) of volumes below the motion threshold. Whole-brain analyses were then conducted using all available subruns. Fig. 3 shows responses to scenes > all control conditions in a group random effects analysis using the full sample (N = 32 subjects; Fig. 3*A*) as well as in individual infants (Fig. 3*B*). At low thresholds (*P* < 0.05, uncorrected), greater responses to scenes than control conditions are found in areas consistent with adult scene regions, while a preference for the control conditions is found in areas consistent with adult object and face regions. Greater responses to scenes than control conditions were also detected (at this uncorrected threshold) in areas outside the traditional scene network, where greater responses to scenes have also been found in adults, including early visual cortex (e.g., refs. 18, 24, and 34–36), cerebellum (e.g., refs. 5 and 35), parietal cortex (e.g., refs. 18, 35, and 37–39), motor/premotor cortex (35, 37) and frontal cortex (e.g., ref. 40). Similar results were found when comparing whole-brain responses to scenes vs. each individual control condition (*SI Appendix*, Fig. S2) and after applying additional spatial smoothing to individual infant data prior to group random effects analysis, to help account for imperfect registration between infant brains (*SI Appendix*, Fig. S3). Group-level activation in infant scene regions was not significant after correcting for multiple comparisons. Further analyses of the number of infants with significant activation and the number of significant voxels per infant are included in the *SI Appendix*, *Supplemental Materials*.

To test the functional selectivity of these regions more directly and stringently, we next conducted subject-specific functional region of interest (ssfROI) analyses. These analyses use one part of the data (i.e., one contrast in n − 1 subruns) to identify each functional region in each individual infant, and then test that region’s response profile in independent data (the nth subrun, iterated across subruns) from the same infant and session. ssfROI analyses are more sensitive than group analyses as they account for subject-specific anatomical differences and do not require correcting for thousands of comparisons across voxels (41–43). Because independent partitions of data are used to identify the ssfROI vs. to test the response to experimental conditions, these analyses are unbiased, and are the gold-standard method for determining the selectivity of functional regions in cortex.

**Fig. 4.**
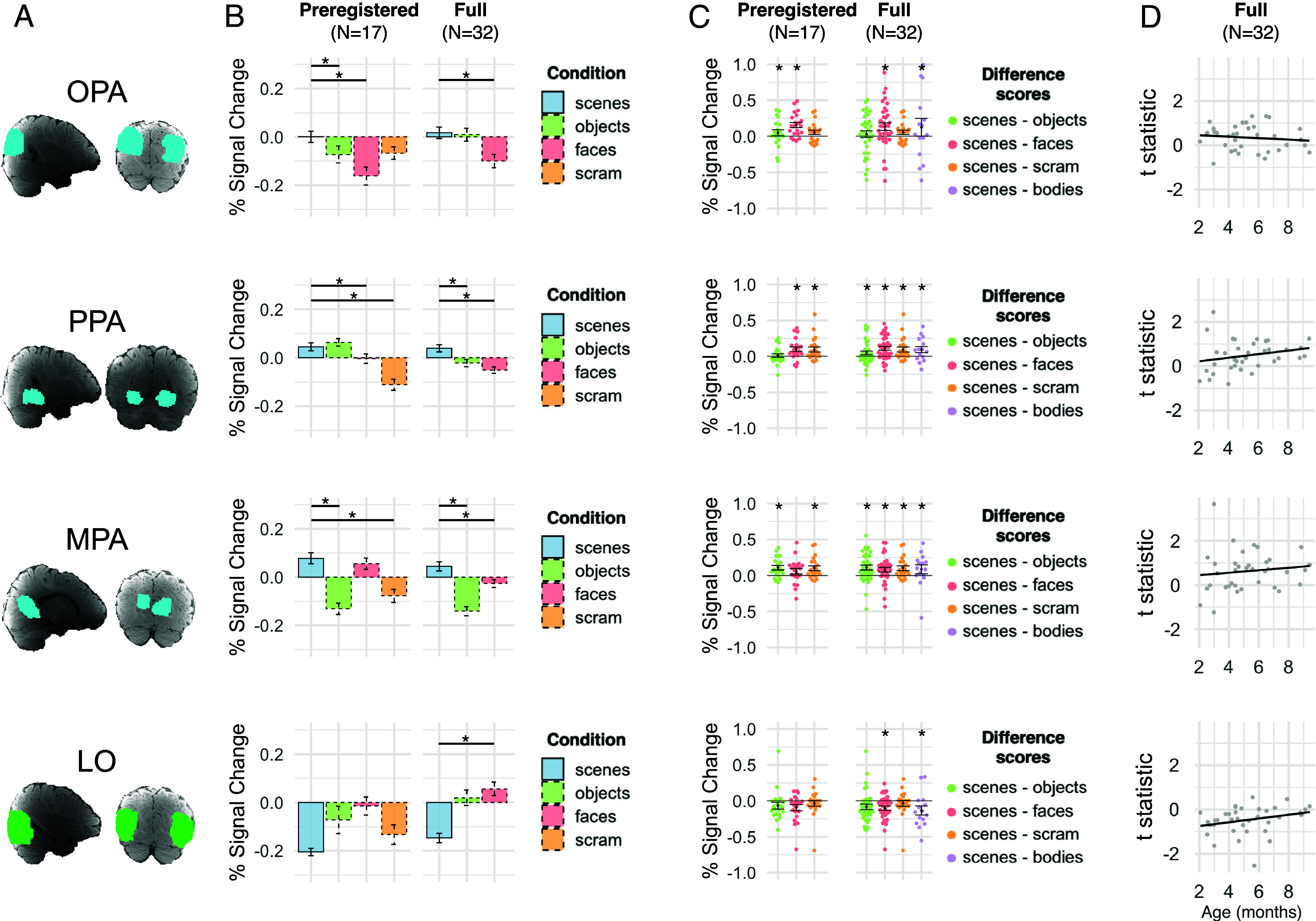
Scene-selective cortex is detectable in 2 to 9-mo old infants and functionally dissociable from responses in nearby object-preferring cortex. (*A*) Search spaces for the OPA (*Top* row), PPA (*Second* row), and MPA (*Third* row) are indicated in teal, and the search space for LO is indicated in green. (*B*) The *Left* set of bar plots indicate results from the preregistered sample (N = 21 sessions from 17 infants), while the *Right* set of bar plots indicate results from the full sample (N = 36 sessions from 32 infants). Bar plots depict the mean response to scenes (blue), objects (green), faces (red), and scrambled scenes (orange). Dashed lines on control conditions reflect the fact that these conditions were sampled 33.3% as often as the scene condition (in the preregistered dataset only). For the full dataset, only conditions sampled in all 32 infants (i.e., scenes, objects, and faces) are depicted. Lines with asterisks indicate the response to scenes was significantly greater than the response to the respective control condition in the linear mixed effect model controlling for age and head motion (*P < 0.05). (*C*) Difference plots for the response to scenes minus each control condition. The response to scenes minus objects is plotted in green; the scene response minus the face response is plotted in red; the scene response minus scrambled scene response is plotted in orange; and the scene response minus the body response is plotted in purple. Colored dots indicate individual participants. Note there are fewer points for scenes-minus-scrambled and scenes-minus-bodies because responses to scrambled scenes were only measured in the preregistered dataset, and responses to bodies were only measured in the Kosakowski et al. (26) dataset. Asterisks indicate the response to scenes was significantly greater than the response to the respective control condition in the linear mixed effect model controlling for age and head motion (*P < 0.05). (*D*) Each plot depicts the difference in response to scenes minus the average of the control conditions as a function of age (in months), using the full sample of infants. Gray dots indicate individual infants. No region showed a significant effect of age. All error bars indicate SEM corrected for within-subject variability (45).

In this case, ssfROIs were defined within previously described anatomical parcels, or “search spaces,” that capture the typical spatial locations of the OPA, PPA, and MPA in adults (44) (Fig. 4*A*). Given the imperfect registration of adult anatomical templates to infant functional data, each parcel was increased in size, using a similar approach as Kosakowski et al. (26). ssfROIs for scene regions were defined as the top 5% of voxels in each parcel responding to scenes > control stimuli (the average of faces, objects, and scrambled for the preregistered sample, or faces, objects, and bodies for the Kosakowski sample). The 5% threshold was preregistered, following prior infant studies (26).

Results of the ssfROI analysis are shown in Fig. 4. We began by comparing responses to scenes vs. the average of all control stimuli in each infant scene region. For the preregistered sample, we found greater responses to scenes than control stimuli in OPA (β = 0.05, t_(61.47)_ = 2.38, *P* = 0.01), PPA (β = 0.03, t_(65.48)_ = 2.31, *P* = 0.01) and MPA (β = 0.04, t_(60.58)_ = 2.85, *P* = 0.003), with no significant region × condition interaction for any pair of scene regions (all F’s < 0.58, all *P*’s > 0.57). Similar results were found for the three scene regions in exploratory analyses of the full sample (all β’s > 0.042, all t’s_(109.37)_ > 2.23, all *P*’s < 0.02; no significant region × condition interaction for any pair of scene regions: all F’s < 0.02, all *P*’s > 0.90).

To provide a stricter test of scene selectivity, we also compared responses to scenes with each individual control condition (noting that we had less power to detect these effects, given that each control condition was presented 1/3 as often as the scene condition in the preregistered dataset). For the preregistered dataset, an exploratory omnibus analysis considering data from all three scene regions (with a factor for region) revealed greater responses to scenes than each of the three control conditions (all β’s < −0.07, all t’s_(223.58)_ < −2.24, all *P*’s < 0.02). Similarly, for the full dataset, this analysis (including an additional factor for experiment) revealed greater responses to scenes than to each of the four control conditions (all β’s < −0.07, all t’s_(392.23)_ < −2.71, all *P*’s < 0.004). The same results were found when accounting for additional indicators of data quality (i.e., after including additional covariates of total volumes per infant and variance explained by the GLM; preregistered sample: all β’s < −0.07, all t’s_(223.67)_ < −2.23, all *P*’s < 0.02; full sample: all β’s < −0.07, all t’s_(390.59)_ < −2.71, all *P*’s < 0.004). Results for these comparisons tested separately in each scene region are summarized in Fig. 4 *B* and *C* and detailed in the *SI Appendix*, *Supplemental Materials*. Additional sliding window analyses (e.g., ref. 26) confirmed that scene-selective responses are robust to analytic parameters for voxel selection, and provide estimates of the size of scene-selective activation in each region (*SI Appendix*, Fig. S4). Further control analyses found that scene selectivity was detected more strongly in scene areas than nearby parcels for face-selective areas [while faceIselective areas responded selectively to faces, replicating previous findings (24, 26, 46)] (*SI Appendix*, Figs. S5 and S6). Taken together, these findings suggest that scene selectivity is present throughout the cortical scene network in infants.

### Infant Scene Cortex Is Functionally Dissociable from Nearby Object-Preferring Cortex.

By adulthood, scene areas are functionally distinct not only from face-selective areas (which are perhaps the most visually-distinct class of stimulus from scenes) but also from nearby areas sensitive to object shape (e.g., refs. 47 and 48). To test for this division of labor in the infant cortex, we compared the functional profile of the infant scene regions to a nearby area in lateral occipital cortex (LO) sensitive to objects (i.e., any stimulus with a visual contour, including objects and faces, but not intact or scrambled scenes).

LO is typically defined based on responses to objects vs. scrambled objects (49, 50). However, we did not measure responses to scrambled objects in either dataset, and therefore opted for the contrast of objects > scenes (*SI Appendix*, *Supplemental Note 1*). LO responses to each condition are shown in Fig. 4. To test for a basic object preference in this region, we conducted exploratory analyses using linear mixed effects models with a factor for objects (i.e., objects, faces, bodies) greater than nonobjects (i.e., scenes, scrambled scenes). This analysis revealed greater responses to objects than nonobjects in the full dataset (β = 0.04, t_(110.2)_ = 2.55, *P* = 0.01), but not the preregistered dataset (β = 0.03, t_(64.51)_ = 1.40, *P* = 0.17). Importantly, this functional profile in LO was functionally distinct from the observed pattern of responses in the scene regions. Analysis of both the preregistered and full datasets revealed a significant region × condition interaction for each scene area compared to LO (preregistered: all F’s_(3,140)_ > 2.70, all *P*’s < 0.05; full: all F’s_(4,243)_ > 4.57, all *P*’s < 0.002), except MPA vs. LO in the preregistered dataset (F_(3,140)_ = 1.83, *P* = 0.14).

Scene-selective cortex is therefore functionally dissociable from nearby object regions within the first 9 mo of life. Findings of three distinct functional profiles also suggest that the pattern of responses in infant scene cortex cannot be explained by general differences in attention, arousal, or basic visual properties (e.g., eyes open vs. closed, luminance) across the experimental conditions.

### Scene Responses Develop Prior to the Onset of Independent Navigation.

**Fig. 5.**
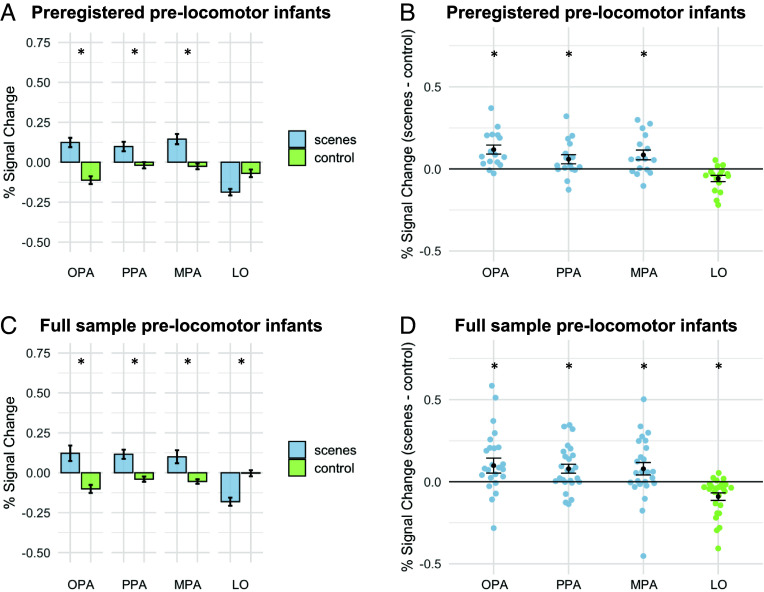
Scene responses are present in prelocomotor infants. (*A*) Data from the preregistered sample of prelocomotor infants only. Bar plots depict the mean response to scenes vs. the average of the control conditions (objects, faces, and scrambled scenes). Error bars indicate SEM corrected for within-subject variability (45). (*B*) Difference score plots for the response to scenes minus the mean of the control conditions. Error bars indicate SEM. Colored dots indicate individual subjects. (*C* and *D*) Same as *A* and *B*, respectively, but now plotting data from the full sample of prelocomotor infants. Asterisks indicate significant results from the linear mixed effect model controlling for age and head motion (*P < 0.05).

Is active experience using scene information for self-guided locomotion required for the development of scene selectivity? If the development of scene selectivity does *not* depend on active navigation experience, then scene regions could be present even in prelocomotor infants. To test this prediction, we analyzed the sample of N = 16 sessions from 13 infants from the preregistered dataset who had never independently locomoted at the time of acquisition (by crawling, walking, or any other means of moving independently to a location more than 3 feet away, per parent report at the time of scan; mean age at time of session = 4.70 mo, range = 2.07 to 7.89 mo). We compared responses to scenes vs. the average of all control stimuli in each infant scene region (Fig. 5). This analysis revealed greater responses to scenes than the control conditions in OPA (β = 0.06, t_(44.93)_ = 3.10, *P* = 0.002), PPA (β = 0.03, t_(49.28)_ = 1.96, *P* = 0.03), and MPA (β = 0.04, t_(46.94)_ = 2.81, *P* = 0.004).

Given the limited sample size of precrawling infants in the preregistered dataset, we also analyzed the full sample of infants. Locomotor status was not assessed directly for infants in Kosakowski et al. (26). Accordingly, for these infants, crawling status was estimated using our EMA data. We identified the youngest age at which any infant in the EMA sample (N = 222) was reported to be able to locomote: age 4.83 mo. There were 9 infants younger than 4.83 mo old in the Kosakowski et al. (26) dataset, yielding a total sample of 25 sessions from 22 prelocomotor infants (mean age at time of session = 4.46 mo, range = 2.07 to 7.89 mo). Analysis of this larger prelocomotor sample revealed significantly greater responses to scenes than control conditions in OPA (β = 0.06, t_(74.34)_ = 2.61, *P* = 0.005), PPA (β = 0.04, t_(76.23)_ = 3.02, *P* = 0.002) and MPA (β = 0.04, t_(74.40)_ = 2.77, *P* = 0.004; responses to individual control conditions are also shown in *SI Appendix*, Fig. S7).

Taken together, these results show that scene-selectivity emerges throughout the scene network prior to locomotor experience.

### Representation of Low-Level Visual Features in Infant Scene Cortex.

Given that infants gain considerable exposure to visual scene statistics even prior to locomotor onset, as confirmed in the EMA study above, we next tested predictions of the passive exposure framework, beginning with the prediction that early-emerging scene responses are explained by low-level visual features.

If low-level features differ systematically between the scene condition and control conditions, then greater responses to scenes should also be found in areas of early visual cortex (EVC) that represent these low-level features. For example, studies of adults have shown greater responses to scenes than control conditions in early visual cortex, with similar patterns found for phase-scrambled stimuli, suggesting these responses can be explained by confounded low-level stimulus properties (51, 52). Consistent with these findings from adults, an exploratory ssfROI analysis revealed that a portion of EVC responded significantly more to scenes than control conditions, both in the preregistered (β = 0.06, t_(59.45)_ = 2.80, *P* = 0.007) and full datasets (β = 0.09, t_(109.48)_ = 3.88, *P* < 0.001) (*SI Appendix*, Fig. S8*A*). To investigate whether scene responses in the infant scene regions are dissociable from this portion of EVC, we conducted an exploratory analysis testing whether scene selectivity is still detectable in infant scene regions after regressing out the signal from the scene-preferring portion of EVC (i.e., by including the mean time course of EVC voxels as a nuisance regressor in the GLM for each subrun). Given that the scene-preferring EVC region is plausibly a key source of visual input to the infant scene regions, and given that timecourses between these nearby regions are likely to be correlated (reflecting both shared signal and noise), this analysis constitutes a high bar for evidence of distinct representations in the infant scene regions vs. EVC. Critically, although scene selectivity was now weaker, the infant scene regions still showed an overall greater response to scenes than control conditions in the full dataset (β = 0.02, t_(823.87)_ = 2.16, *P* = 0.02), although this effect did not reach significance in the preregistered dataset (β = 0.01, t_(472.64)_ = 1.24, *P* = 0.12) (*SI Appendix*, Fig. S8*B*). These results suggest that selectivity in infant scene regions is partially, but not fully explained by information inherited from EVC.

The analysis above depends on reverse inference about infant EVC representation, and cannot reveal the particular features that potentially drive infant scene responses. We next tested whether low-level features known to drive responses in adult scene regions explain responses in infant scene cortex. Specifically, we quantified the mean high spatial frequency (53), rectilinearity (54, 55), and (peripheral) motion energy (56, 57) for each of our stimulus categories using standard approaches (*Methods* and *SI Appendix*, Fig. S9). Given that infants may have looked anywhere in our stimuli, we measured motion energy both in the entire video, and also specifically in the video periphery. We then fit exploratory linear mixed effect models with two factors: a categorical model of scene selectivity (i.e., with scenes coded as 1 and all other conditions as −1) and a low-level feature model (i.e., the normalized mean values of each feature across conditions, tested individually against the category model). To maximize power, we included data from all scene regions (results from individual regions are reported in the *SI Appendix*, *Supplemental Materials*).

For the preregistered dataset, category explained significant variance even after accounting for each of the four low-level feature models (all β’s > 0.07, all t’s_(226.89)_ > 2.81, all p’s < 0.006). No low-level feature explained significant variance after accounting for category (all β’s < 0.02, all t’s_(226.89)_ < 0.95, all *P*’s > 0.34). Collinearity was minimal between the low-level feature models and the category model (correlation of fixed effects ranged from −0.25 to −0.32). The same pattern of results was found in the full dataset, with a significant effect of category after accounting for each low-level feature (all β’s > 0.07, all t’s_(351.76)_ > 3.07, all *P*’s < 0.003), but no significant effect of any low-level feature after accounting for category (all β’s < 0.02, all t’s_(351.74)_ < 1.22, all *P*’s > 0.22). Collinearity was moderate between the low-level feature models and the category model (correlation of fixed effects ranged from −0.56 to 0.01). Additional analyses of a) low-level feature information tested in the nonscene control conditions only and b) combinations of low-level features are reported in *SI Appendix*, *Supplemental Materials*.

Taken together, these analyses provide partial support for the passive exposure framework, insofar as greater responses to scenes than control conditions are also found in early visual cortex, and estimates of scene selectivity in scene regions are weaker after accounting for the signal from this early visual region. However, the results also suggest that scene selectivity in 2 to 9 mo old infants is not entirely explained by low-level features, since scene responses remained after accounting for the signal from early visual cortex, and were not significantly related to motion energy, high spatial frequency, or rectilinear content in the stimuli. One possibility is that infant scene regions, like adult scene regions (58), represent a mixture of both low- and high-level visual features associated with scenes.

### Testing Age-Related Change.

Another key prediction from the passive exposure framework is that scene-selective responses develop gradually with age through accumulating exposure to visual scene statistics across months if not years. Surprisingly, however, we found no reliable evidence that scene selectivity changes with age in any of the three regions (preregistered sample: all β’s < 0.10, all t’s_(16)_ < 0.56, all *P*’s > 0.34; full sample: all β’s < 0.17, all t’s_(31)_ < 1.35, all *P*’s > 0.09) (Fig. 4*D*). Details and additional analyses are provided in *SI Appendix*, *Supplemental Materials*. Given that fMRI estimates of scene selectivity in individual, awake infants are noisy, we emphasize that the lack of evidence of age-related change should be interpreted with caution. Several prior awake infant fMRI studies have also failed to find clear evidence of age-related change in visual responses [(26, 59–61); but see refs. 46, 62, and 63].

We also tested whether scene-selectivity is detectable even in the youngest infants alone. To do so, we median split the full sample of sessions based on age at acquisition, yielding a sample of 19 sessions from only the 18 younger infants (mean age = 3.72 mo, range = 2.07 to 4.93 mo). An exploratory omnibus analysis (across regions, with a factor for region) found greater responses to scenes than the average of the control stimuli (β = 0.04, t_(206.78)_ = 2.64, *P* = 0.004). Stronger responses to scenes than the average of the control conditions were also found in exploratory analyses of OPA, PPA, and MPA tested individually (all β’s > 0.03, all t’s_(56.76)_ > 1.81, all *P*’s < 0.03) (additional analyses of responses to individual control conditions are provided in *SI Appendix*, Table S2). Taken together, these findings suggest that scene-selective responses emerge as early as three and a half months old, corresponding to 286 h of ego-motion, and 147 h of forward-facing ego-motion (almost all of it passive), as estimated using our EMA dataset.

## Discussion

Here we found that the basic signature of scene selectivity emerges in all three known regions of the cortical scene processing network, in similar locations to adults, within the first 9 mo of life. Responses in infant scene regions were i) higher for scenes than all tested control conditions, ii) dissociable from those in nearby face-selective and object-preferring cortex, and iii) partially, but not fully explained by correlated low-level visual properties. Greater responses to scenes than control conditions were observed even in the subsamples of precrawling infants and infants less than 5 mo old. The earliest signatures of human cortical scene selectivity therefore do not require more than a few weeks of passive visual experience and do not require any active experience with independent navigation to develop.

These findings build on prior awake infant fMRI studies showing preferential responses to scenes vs. other categories, particularly in the PPA (24, 26), by revealing that selective responses are present across all three regions of the cortical scene network in infancy. Notably, our claim that basic scene selectivity emerges across the scene network in infancy does not imply that the magnitude of scene selectivity is fully adult-like (*SI Appendix*, *Supplemental Note 2*). Nevertheless, these findings add to growing evidence that the functional organization of the infant visual system is already similar to that in adults. Within the first year of life, early visual cortex is retinotopically organized (59, 60), while higher-level visual cortex includes areas selective not only for scenes but also for faces (24, 26, 46, 61), bodies (26), and motion (62, 64), as well as broad tuning for semantic dimensions (e.g., animacy, real-world size) not easily explained by low-level visual features (63).

We speculate that the success of the current study in finding scene-selective responses in all three regions may be attributed to the increased size of the sample of data collected using the newer custom head coil (65) and higher-resolution EPI sequences, relative to the datasets used in our previous studies (24, 26). Another possibility is that aspects of the current design, such as using dynamic stimuli that maximally activate scene regions in adults, might have contributed to our success–although we found no reliable difference in scene selectivity between the preregistered and Kosakowski et al. (26) datasets. Combining the two samples increased the statistical power of our analyses, and revealed significantly higher responses to scenes than each of four control conditions tested (objects, faces, scrambled scenes, bodies) in all three regions. In the smaller preregistered sample alone, a few of these comparisons were nonsignificant trends, confirming the value of a larger sample.

The presence of scene-selectivity in young infants places new constraints on the passive exposure framework for the development of category-selective visual areas (66). If passive exposure drives the initial development of scene responses, this process must occur within the first ~100 d of visual experience (based on the median age of our younger infant sample), which we estimate includes 286 h of ego-motion, and 147 h of forward facing ego-motion, almost all of it passive. Recent computational modeling suggests that such a rapid timeline is plausible: For example, DNNs trained with just 200 h of head-camera footage from a single child achieve 70% of the accuracy of a high-performance ImageNet-trained model (67). However, this work sampled only older infants’ visual diets and did not account for the limited nature of very young infants’ vision. In the first 5 mo, many relevant visual processes are still immature, including visual acuity (and thus sensitivity to high spatial frequencies and rectilinearity), peripheral visual input, depth and distance perception, and oculomotor control (68, 69). Accordingly, our results are also consistent with the hypothesis that little to no visual exposure is necessary to support the initial emergence of scene selectivity, consistent with evidence of scene responses in the congenitally blind (70). The finding that scene regions are functionally dissociable from nearby object and face regions early in infancy is consistent with evidence that connectivity underlying high-level visual areas may be present at or before birth, potentially guiding the development of category selectivity from early on (8, 71–74).

Our findings of scene selectivity in prelocomotor infants also rule out a prediction of the active experience framework: that active experience with independent locomotion is necessary for the initial development of scene selectivity. This finding is consistent with but more definitive than prior observations of scene-selective fMRI and fNIRS responses in the infant brain (24–26), as well as EEG studies showing distinct responses to scenes vs. other conditions in infancy (27, 28).

Importantly, while these results constrain both the passive exposure and the active experience frameworks, they do not rule out a role for either passive exposure and/or active experience in the subsequent development of scene representations. Passive exposure may support a gradual process of refinement of scene representations across years of childhood visual experience (e.g., ref. 75), although existing evidence for age-related change in scene regions across childhood is somewhat inconsistent (e.g., refs. 17–23, 35, 76, and 77). Active experience may play an important role in strengthening or maintaining scene selectivity later in infancy, and in refining the more specific scene representations extracted by these regions. Growing evidence suggests that by adulthood, OPA, PPA, and MPA extract different representations and serve distinct functional roles within scene processing (3, 78). While we measured only general scene selectivity, a functional property shared among the three scene regions, the finding that all three regions are detectable in infancy opens the door for future research testing whether more specific divisions of labor are also present from early on, or only arise later and increase gradually across childhood (e.g., refs. 19, 76, 77, 79, and 80). Future work could also explore whether experience matters more for some regions than others, and importantly, the specific kind of experience (e.g., active experience vs. passive exposure) that is necessary for further specialization to unfold. In any case, our results highlight the potential of cortical scene processing as a test case for understanding the relative roles of active experience and passive exposure in shaping the development of the human brain

In conclusion, here we show that scene selectivity is present throughout the cortical scene network in infancy, earlier than previously observed, and prior to experience with independent navigation. These findings provide a constraint on theories of how these regions develop, and more broadly highlight the potential of cortical scene processing as a test case for understanding the relative roles of active experience and passive exposure in shaping the development of the human brain.

## Methods

### Participants.

Subjects were healthy human infants ages 2 to 9 mo old. This age range was chosen based on our success in recruiting and collecting data from similarly aged infants in prior awake infant studies (24, 26, 46). Participants were recruited via online ads posted on social media sites (Facebook and Instagram), local posters, and word of mouth. The final sample size depended on several thresholds for inclusion based on data quality and quantity, outlined in detail below. Infants were invited to return for multiple sessions; sessions conducted less than 31 d apart were treated as a single session and analyzed together, while sessions conducted more than 31 d apart were analyzed separately. For the preregistered dataset, we conducted 102 visits with 64 infants. After applying data inclusion thresholds and collapsing across sessions, we obtained a final sample size of 21 sessions from 17 infants (mean age at time of session = 5.40 mo, range = 2.07 to 9.11 mo). For the full sample, we additionally analyzed the subset of infants from Kosakowski et al. (26) that were scanned using the same head coil and sequence used for the preregistered sample. Full data retention information for the infants from Kosakowski et al. is provided in the associated manuscript (26). Including these additional infants lead to a full sample of 36 sessions from 32 infants (mean age at time of session = 5.25 mo, range = 2.07 to 9.41 mo). Full subject and session information are provided in *SI Appendix*, Table S3. The scanning success rates in these datasets are typical for awake infant fMRI, after accounting for the strict head motion thresholds applied here (81). In addition to the fMRI experiment, parents of infants in the preregistered sample only completed an in-lab demographic questionnaire, as well as a subset of twelve questions taken from the Peabody Developmental Motor Scales, selected to identify experience with independent navigation, from belly crawling to walking with and without support. Parents were also asked to report directly whether their infant has ever successfully crawled or walked. Specifically, we asked parents whether, and if so, how their child is capable of locomoting toward a goal – by any means, including any form of crawling – more than three feet away. For navigating infants, we further collected a calendar date of crawling or walking onset (per parent report). Parents of infants gave informed consent prior to participation. Ethical approval for this research was obtained through the Internal Review Board at MIT.

### Stimuli.

Stimuli for the preregistered dataset are available at https://osf.io/vk7aw/. Stimuli for the Kosakowski et al. (26) dataset are available at https://osf.io/jnx5a/, and are described in detail in the paper. For the preregistered dataset, 60 scene stimuli were chosen based on the results of a pilot fMRI experiment (*SI Appendix*, *Supplemental Materials*). Infants also viewed stimuli from three control categories to provide a test of scene-selectivity, including 20 object stimuli (taken from refs. 37 and 82), 20 face stimuli (taken from ref. 82), and block-scrambled versions of the top 20 scene stimuli. To further attempt to maximize BOLD responses and increase infant attention, each video was shown for 2.7 s, followed by a 300 ms still image from the same category (but not taken from the same videos), following the design of Kosakowski et al. (26, 46). Scene, face, and object still images were taken from a variety of sources (26, 37, 47, 83), and scrambled scene still images were created from the still scene images using the same block-scrambled approach. Additional details regarding stimulus selection and low-level visual feature analyses are provided in the *SI Appendix*, *Supplemental Materials*.

### Design.

Stimuli were presented in a block design at 13.7 × 18.1 degrees visual angle, with six 3 s video + still image pairs presented per block, yielding 18 s blocks. For only the preregistered dataset, the block sequence alternated between scenes and control blocks, such that scene blocks were shown every other block, and the three control blocks were shown in a pseudorandom sequence such that all three control conditions were always shown before any scene condition was repeated. This design was chosen to ensure adequate sampling of scene activation in any given subrun. A block of colorful attention-getter object stimuli, with accompanying sounds, was also shown every 8 blocks. A run began as soon as the infant was in the scanner and videos played continuously for as long as the infant was engaged and awake. If infants fell asleep, we continued collecting resting state data for at least 10 additional minutes. Similar methods were used for the Kosakowski et al. (26) dataset, except that scene blocks were presented in approximately equal proportion to control blocks, and the baseline condition consisted of curvy, abstract movies rather than attention getters with accompanying sounds. Potential differences in baseline activation between the two datasets were accounted for via a factor for experiment in all linear mixed effects models of the full dataset.

### Data Collection.

Infants were swaddled if the caregiver thought it would be useful. A parent or researcher went into the scanner with the infant while a second adult researcher (“scanner buddy”) stood outside the bore of the scanner, while maintaining a view of the infant’s face through the bore. The scanner buddy monitored infant attention and indicated to a researcher in the control room when the infant was not looking at the stimuli. The researcher in the control room then recorded these TRs for exclusion during preprocessing. Infants heard lullabies (https://store.jammyjams.net/products/pop-goes-lullaby-15) played through custom infant headphones for the duration of the scan.

### Custom Head Coil and Equipment.

Infants were scanned in a 32-channel head coil designed to comfortably cradle infants while wearing custom infant MR-safe headphones (65). Infant headphones attenuated scanner noises (attenuation statistics reported in ref. 65) and allowed infants to listen to music at a comfortable volume for the duration of the scan. An adjustable coil design increased infant comfort, accommodated headphones, and suited a variety of infant head sizes. The infant head coil and headphones were designed for a Siemens Prisma 3T scanner and enabled the use of a standard trajectory EPI with 43 to 44 near-axial slices (repetition time, TR = 3 s, echo time, TE = 30 ms, flip angle = 90, field of view, FOV = 160 mm, matrix = 80 × 80, slice thickness = 2 mm, slice gap = 0.0 to 0.4 mm).

### Data Selection (Subrun Creation).

To be included in the analysis, data were required to meet the criteria for low head motion that was reported by Deen et al. (24) and Kosakowski et al. (26). Data were cleaved between consecutive time points having more than 2 rad or 2 mm of motion, creating subruns which contain at least 24 consecutive low-motion volumes. All volumes included in a subrun were extracted from the original “run” data (a run began when the subject went into the scanner and ended when the subject fell asleep or became fussy) and combined to create a new NIfTI file for each subrun. Event files were similarly updated for each subrun. Volumes with greater than 0.5 rad or mm of head motion between volumes were scrubbed. To be included in the ssfROI analysis, subjects were required to have at least two usable subruns of length 96 vol. each (one to choose voxels based on the relevant contrast, and the other to independently extract response magnitudes from the selected voxels). We originally preregistered a lower threshold for data inclusion, requiring just two usable subruns of 24 vol. each. However, upon analyzing the data from Kosakowski et al. (26) in a pilot analysis focused on responses in the infant fusiform face area, we found that inclusion of infants with such low data quantity led to noisier selectivity estimates of face selectivity. We therefore chose to require 96 vol. in each split-half of the data, following the approach used in Kosakowski et al. (26). For subjects with only one subrun, the originally selected subrun was split in half (discarding the middle two volumes), and each half was reevaluated for meeting the subrun criteria described above. If both halves were usable, the subrun was split; if not, the subject was discarded.

### Preprocessing.

Each subrun was processed individually. First, an individual functional image was extracted from the middle of the subrun to be used for registering the subruns to one another for further analysis. Then, each subrun was motion corrected using FSL MCFLIRT. If more than three consecutive images had more than 0.5 mm or 0.5 rad of motion, there had to be at least seven consecutive low-motion volumes following the last high-motion volume in order for those volumes to be included in the analysis. Additionally, each subrun had to have at least 24 vol. after accounting for motion and sleep TRs. Functional data were skull-stripped (FSL BET2), intensity normalized, and spatially smoothed with a 3 mm FWHM Gaussian kernel (FSL SUSAN). Information on registration is provided in *SI Appendix*, *Supplemental Materials*.

### Parcels/Search Spaces.

Group constrained parcels were acquired from previous adult research to localize areas of selectivity (44). Scene-selective parcels included OPA, PPA, and MPA, the object preferring parcel was LO, and face-selective parcels included OFA, FFA, STS, and MPFC. All parcels included separate left and right hemisphere parcels. Given the difficulty of obtaining reliable registrations with infant functional data, we dilated the OPA parcel to increase the chances of capturing peak voxels in each region. The final parcels are available on OSF. SsfROIs were defined as the top 5% of voxels within each search space. Following this procedure, the average size (given minor differences between subjects due to registration error) of the final ssfROIs were as follows: OPA = 183 voxels, PPA = 63 voxels, MPA = 74 voxels, and LO = 252 voxels.

### Subject-Level Beta and Contrast Maps.

Functional data were analyzed with a whole brain voxel-wise general linear model (GLM) using the FSL software. The GLM included four condition regressors (scenes, objects, faces, and scrambled scenes), 6 motion regressors, a linear trend regressor, and 5 PCA noise regressors. Condition regressors were defined as a boxcar function for the duration of the stimulus presentation (18 s blocks). Infant inattention or sleep was accounted for using a single impulse nuisance (“sleep”) regressor. The sleep regressor was defined as a boxcar function with a 1 for each TR where the infant was not looking at the stimuli, and the corresponding TR for all condition regressors was set to 0. Boxcar condition and sleep regressors were convolved with an infant hemodynamic response function (HRF) that is characterized by a longer time to peak and deeper undershoot compared to the standard adult HRF (84). Where required for ssfROI analyses, data and regressors were concatenated across subruns, run regressors were added to account for differences between runs, and beta values were computed for each condition in a whole-brain voxel-wise GLM. The GLM was fit separately for splits of the runs in each fold of the leave-one-out cross-validation procedure, ensuring estimates were independent in the data used to define vs. test responses. PCA noise regressors were computed using a method similar to GLMDenoise (85) as defined in Deen et al. (24). Specifically, we first defined a “noise pool” of voxels whose activation was unrelated to the experimental paradigm, based on the results of an initial GLM including condition and motion impulse regressors, and PCA was then run on the timecourses from these noise pool voxels, in order to extract time courses for the top 5 principal components, which were then included as nuisance regressors in the GLM. Noise pool voxels were defined as the bottom 1% of voxels showing the weakest activation to the experimental conditions, calculated as the sum of the z-scored betas for the experimental conditions. For ssfROI analyses, percent signal change was calculated by normalizing the parameter estimates for each condition relative to the mean signal intensity in each voxel.

### Group Random Effects Analysis.

To test whether there was systematic overlap between areas of activation across infants, we conducted group random effects analyses using the full sample of infants from the ssfROI analysis (N = 32 infants). For each session, we generated a single activation map using the data from all available subruns, and for subjects with multiple sessions, we averaged these maps across sessions, yielding a single map per subject. A nonparametric one-sample *t* test was performed across subjects using FSL’s randomise (5000 permutations, TFCE correction) (86). For visualization and reporting purposes, voxelwise statistical maps for the contrast of scenes > all control conditions were transformed to infant template space (see data registration method above).

### Statistical Analyses.

Statistical significance was assessed using using linear mixed effects models (via the lme4 package in R). All models included factors for age (in months, normalized) and head motion (proportion of volumes with greater than 0.5 mm motion across all subruns, per infant), as well as a random effect of subject. The fixed effect of age and random effect of subject allowed us to account for infants who produced usable datasets at two different timepoints. Some analyses also included factors for additional metrics of data quality, including total volumes (sum of usable subrun lengths, per infant) and variance explained by the GLM (1-residual variance divided by total variance, calculated per infant from a GLM including data from all usable subruns), as noted. For analyses including data from more than one region, models included a factor for region. For analyses of the full dataset, models included a factor for experiment.

We preregistered two approaches to test scene selectivity. First, to maximize statistical power to detect a difference in response between scenes and control stimuli, we fit models with a regressor for “scenes_vs_control” with 1’s for scenes and −1’s for all other conditions (objects, faces, scrambled scenes, and/or bodies). The response was deemed selective if the fixed effect coefficient for scenes_vs_control was significantly positive. Second, to provide a stricter test of scene selectivity, we fit models with regressors for each control condition individually. The condition models had 1’s for the corresponding condition (e.g., 1’s for faces in the face regressor) and 0’s for all other conditions. Responses in a region were deemed selective if the fixed effect coefficient for all control conditions was significantly negative. Following our preregistered analysis plan, p values were assessed with one-tailed tests where a specific direction of effect was predicted (e.g., greater responses to scenes than control stimuli; stronger responses in older than younger infants; stronger responses for higher rectilinearity stimuli). Exploratory analyses testing for effects in the same direction as the preregistered analyses were also tested using one-tailed tests. Two tailed tests were used for all other exploratory analyses.

## Supplementary Material

Appendix 01 (PDF)

## Data Availability

Anonymized ssfROI analysis results and whole-brain map data have been deposited in OSF for the preregistered (https://osf.io/vk7aw/ (87)) and Kosakowski et al. datasets (https://osf.io/jnx5a/ (88)). Original fMRI images from infants in the preregistered dataset are available on openneuro.org (89).
