# Supplementary material for: The cortical scene processing network emerges in infancy, prior to independent navigation experience
Source: Proc Natl Acad Sci U S A. 2026 Jul 28;123(31):e2513126123. doi: 10.1073/pnas.2513126123 (PMC13439116; doi:10.1073/pnas.2513126123)
Supplement: Supplementary file 1 [file pnas.2513126123.sapp.pdf]

Supporting Information for

The cortical scene processing network emerges in infancy, prior to independent navigation experience.

Frederik S. Kamps, Emily M. Chen, Haoyu Du, Heather L. Kosakowski, Ariel Fuchs, Nancy Kanwisher, Rebecca Saxe

Corresponding Author: Frederik S. Kamps
Email: fkamps@ed.ac.uk

This PDF file includes:

Supporting text
Figures S1 to S9
Tables S1 to S3
SI References

Stimulus selection

To test whether the new scene videos we selected for the preregistered dataset elicited stronger responses in adults than the stimuli used in our previous infant fMRI experiment (1), in our pilot study we compared the new scenes to a representative sample of 9 stimuli from that experiment ("old scenes"). The top stimuli ("new scenes") were selected and tested using a crossvalidation procedure. Specifically, we defined the top stimuli using data from N-1 subjects, and then measured responses to those stimuli (and the old stimuli) in the left out subject, iterating across all permutations. We found a clear effect, with the new scenes eliciting stronger responses than the old scenes for all five subjects in each of the three regions. Our final stimulus set for infants was chosen based on responses averaged across all 5 adult subjects. The top 60 scene videos were selected based on highest responses in the OPA, although we found no evidence that the stimuli selected differed reliably between regions.

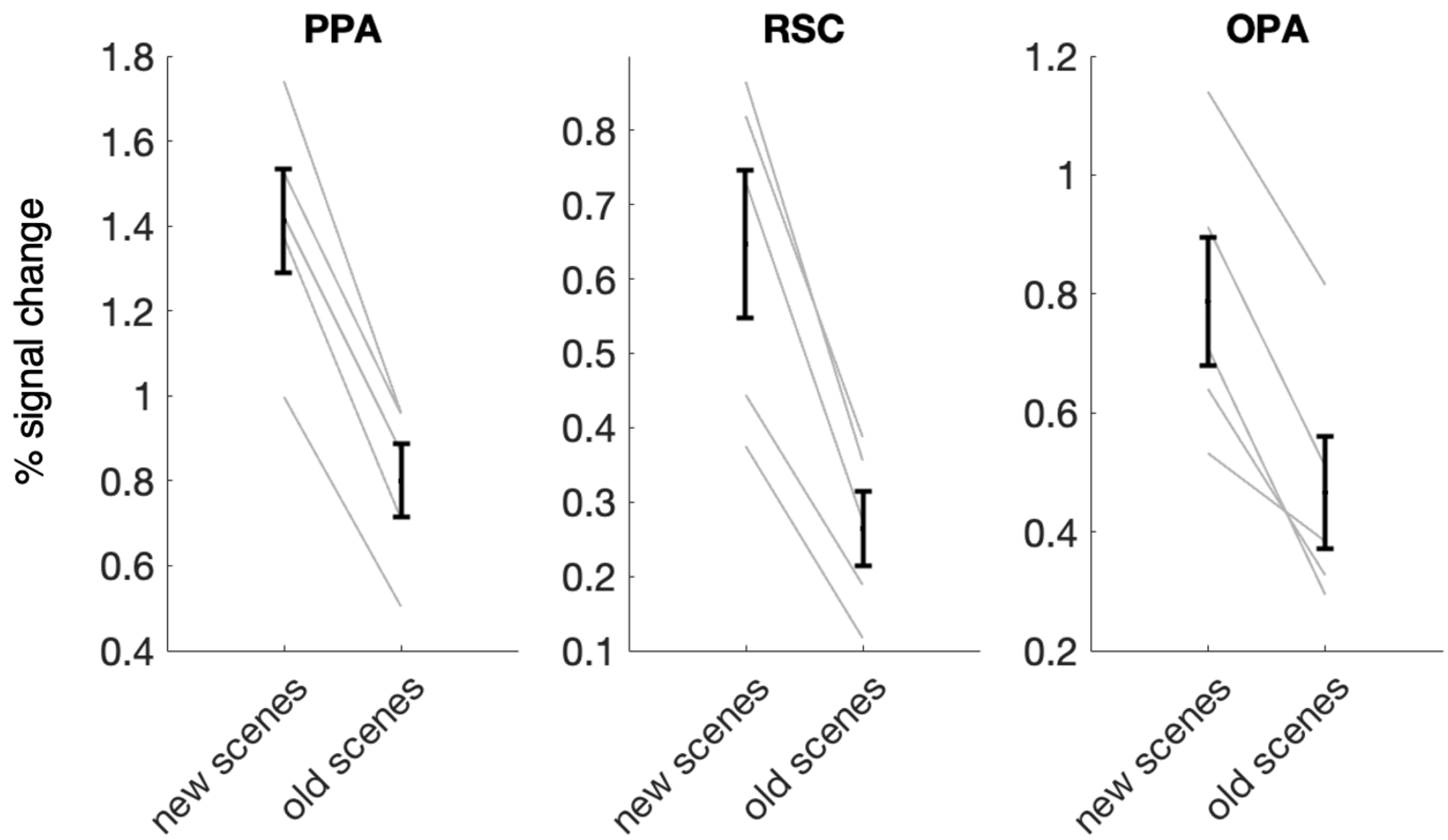

Supplemental Figure 1. Results of pilot adult fMRI study testing responses to the stimuli used in the pregregistered experiment ("new scenes") versus a subset of 9 stimuli used in Kosakowski et al ("old scenes"). Methods are described above and in Chen et al. (2). Error bars indicate standard error of the mean, and grey lines indicate data from individual subjects.

Methods for estimating scene experience

How much experience moving forward through a scene do infants gain in the first two to nine months of life? Using the EMA data, we estimated the amount of experience per day for any given age by multiplying the observed proportions of ego-motion experience responses (i.e., in Main Figure 1A-D) by estimates of the amount of time spent awake per day at each age. Based on prior work and established recommendations for infant sleep (3), we estimated that infant wakefulness increases gradually from 7.5 hrs per day at birth to 10 hrs per day at 9 months. To calculate overall experience with ego-motion through the environment, we multiplied the vector of time spent awake per day by the vector of proportion “yes” ego-motion responses (as a function of age) in Figure 1A. To account for the fact that ego-motion events may differ in length, as a function of age, we further multiplied these vectors by the vector of proportion “yes” responses to ego-motion duration greater than 10 minutes in Figure 1B. Finally, to estimate forward-facing ego-motion experience, we additionally multiplied by the vector of proportion “yes” forward ego-motion responses in Figure 1C. The resultant vectors were interpolated to provide one value per day of life, and cumulative experience was then calculated as the sum of the final vector. These rough estimates from the EMA study do not account for (i) reduction in information by limited acuity and periphery vision (4, 5), or (ii) additional information from very short movements and postural adjustments.

EMA Current Activity Survey Questions

1. Are you with the child right now? [Yes, No]
2. Is the child awake right now? [Yes, No, Uncertain]
3. In the last 5 minutes, was the child ever moving through their environment (e.g., by crawling, walking, being carried, or riding in a stroller or car)? [Yes, No, Uncertain]
4. Think of the most recent time you saw the child awake and moving through the environment. Please briefly describe the event (e.g., “I carried the infant from the bedroom to the kitchen”, or “the infant was crawling around on the living room floor”). [Free Response]
5. Think of the most recent time you saw the child awake and moving through the environment. How long ago was that? [in the last 5 minutes, 5-30 minutes ago, more than 30 minutes ago]
6. Think of the most recent time you saw the child awake and moving through the environment. Was the child controlling their own motion, or was someone/something else? [They controlled their own motion (e.g., by crawling or walking), Someone/something else controlled their motion (e.g., being carried or riding in a stroller or car)]
7. Think of the most recent time you saw the child awake and moving through the environment. Which direction was the child primarily looking while moving? [Forward, in the direction they were moving (e.g., while crawling, or riding in a front-facing stroller); Backwards, opposite the direction they were moving (e.g., in a rear-facing stroller); Uncertain or other]
8. Last question! Think of the most recent time you saw the child awake and moving through the environment. How long was the child moving that way? [For a few seconds, for a few minutes, for ten minutes or more]

Additional group random effects analyses

In addition to our primary group random effects analysis for the contrast of scenes > all conditions, we also conducted similar analyses for contrasts of scenes versus each individual control condition. Note that these contrasts are less powerful than the primary group random effects analysis since they involve only subsets of the data and/or participants.

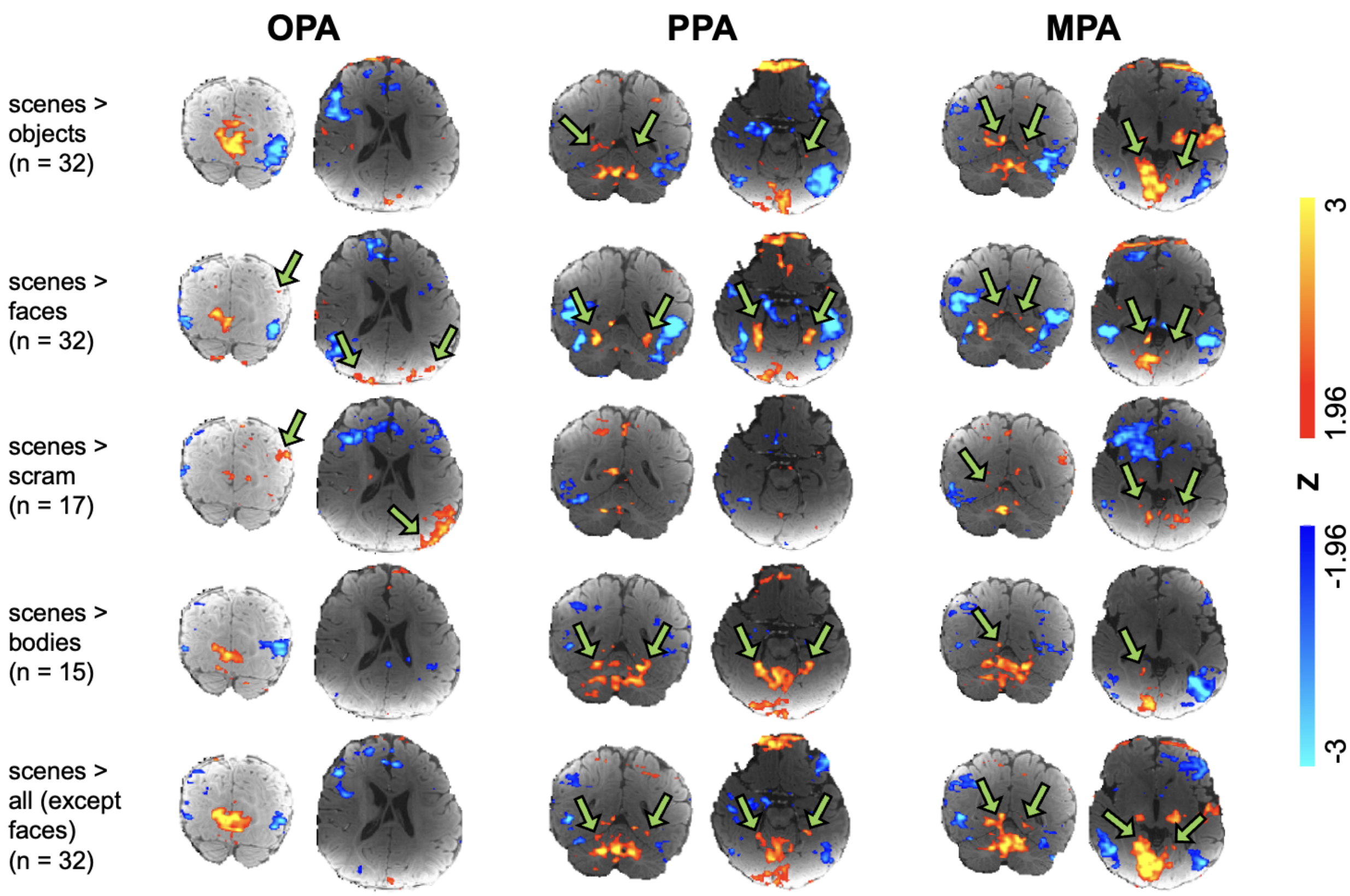

Supplemental Figure 2. Whole-brain activation for contrasts of scenes versus each individual control condition. Regions preferring scenes in warm colors, and regions preferring the control conditions in cool colors. Data are shown at a low, uncorrected threshold ($p < 0.05$; transformed to z for visualization). Activation in scene regions was not significant after accounting for multiple comparisons.

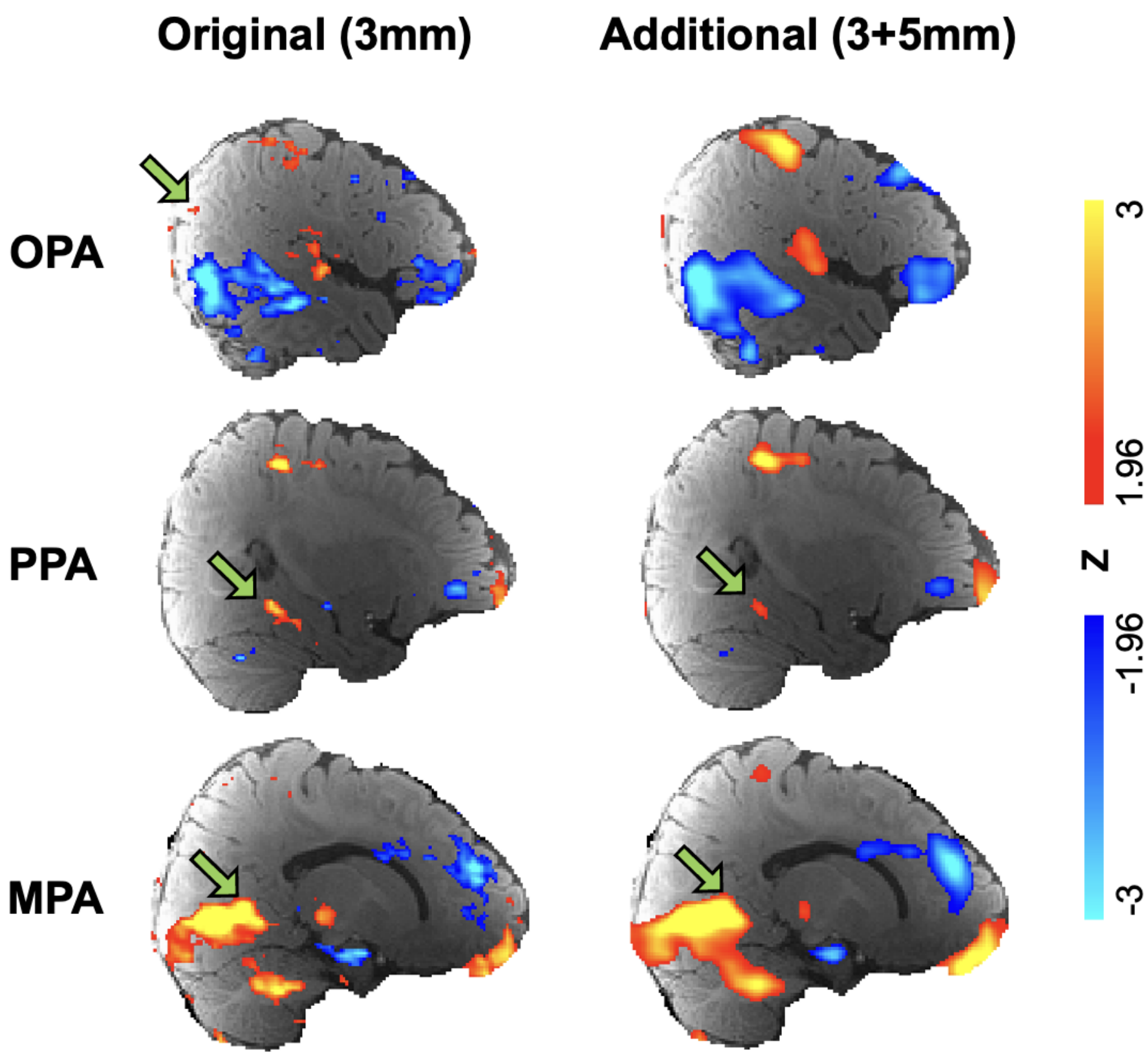

Supplemental Figure 3. *Effects of additional spatial smoothing on group random effects analyses.* Maps show activation for the contrast of scenes vs. the average of all control conditions, with regions preferring scenes in warm colors, and regions preferring the control conditions in cool colors. Data are shown at a low, uncorrected threshold ($p < 0.05$; transformed to z for visualization). The left column shows the original data reproduced from Figure 3A. The right column shows the results for an analysis in which the individual subject maps (initially smoothed by 3mm, following our preregistered analysis plan) were smoothed by an additional 5mm kernel, prior to group analyses. Despite additional smoothing, activation in scene regions was not significant after accounting for multiple comparisons.

Analysis of significant voxels within scene region parcels

To better understand whole-brain activation to scenes in individual brains, we explored how often significant voxels (for the contrast of scenes > all) were detected in the anatomical vicinity of each scene region in each session (N=36). To do so, we overlaid the ROI search spaces on wholebrain maps calculated using all subruns from each session, and calculated the number of significant voxels within each search space. The percentage of infants with at least one significant voxel ($p < 0.05$, uncorrected) was 97% for OPA (mean = 323 voxels, 9% of voxels in search space), 94% for PPA (mean = 68 voxels, 5% of voxels in search space), and 86% for MPA (mean = 65 voxels, 4% of voxels in search space). However, in most sessions, none of these voxels survived correction for multiple comparisons: The percentage of infants with at least one significant voxel after FDR correction ($p < 0.05$, whole-brain correction) was 31% for OPA (mean = 10 voxels), 14% for PPA (mean = 1 voxel), and 6% for MPA (mean = 1 voxel). The number of significant ($p < 0.05$, uncorrected) voxels was not significantly correlated with the number of volumes collected per session in any region (all r's < 0.14, all p's > 0.43). These results suggest that despite our efforts to retain only high-quality data from awake infants, data from individual sessions are generally too noisy to detect strong scene-selective activation surviving multiple comparison correction.

Analyses of scene selectivity in individual scene regions

To further test infant scene selectivity, here we report comparisons of responses to scenes versus each control condition, separately in each region. Note that estimates of the responses to individual control conditions are based on less data, since each control condition was sampled 3x less often than scenes in the preregistered dataset. Note also that the set of conditions differed between the two samples; both included scenes, faces, and objects, while only the preregistered sample included scrambled scenes and only the Kosakowski et al. (1) sample included bodies. Detailed statistical reporting for each contrast of scenes versus each control condition in each region is provided in Supplemental Table 1 below (for visualization, see Figure 4).

Supplemental Table 1

Preregistered dataset

Region	Control contrast	beta	df	t value	p value	p<0.05
OPA	object	-0.1	59.32	-1.85	0.03	*
OPA	face	-0.16	59.32	-3.03	0.002	*
OPA	scram	-0.05	59.32	-1.05	0.15	

Region	Control contrast	beta	df	t value	p value	p<0.05
PPA	object	-0.008	63.41	-0.24	0.41	
PPA	face	-0.1	63.41	-2.91	0.003	*
PPA	scram	-0.1	63.41	-2.82	0.003	*

Region	Control contrast	beta	df	t value	p value	p<0.05
MPA	object	-0.11	58.58	-2.81	0.003	*
MPA	face	-0.06	58.58	-1.53	0.07	
MPA	scram	-0.1	58.58	-2.63	0.005	*

Full dataset

Region	Control contrast	beta	df	t value	p value	p<0.05
OPA	object	-0.06	106.34	-1.15	0.13	
OPA	face	-0.13	106.34	-2.7	0.004	*
OPA	scram	-0.03	106.34	-0.56	0.29	
OPA	bodies	-0.15	106.34	-2.2	0.02	*

Region	Control contrast	beta	df	t value	p value	p<0.05
PPA	object	-0.05	107.2	-1.76	0.04	*
PPA	face	-0.11	107.2	-4.28	0.0002	*
PPA	scram	-0.11	107.2	-3.52	0.003	*
PPA	bodies	-0.07	107.2	-1.88	0.03	*

Region	Control contrast	beta	df	t value	p value	p<0.05
MPA	object	-0.11	103.31	-3.64	0.0002	*
MPA	face	-0.08	103.31	-2.68	0.004	*
MPA	scram	-0.11	103.31	-2.97	0.002	*
MPA	bodies	-0.07	103.31	-1.77	0.04	*

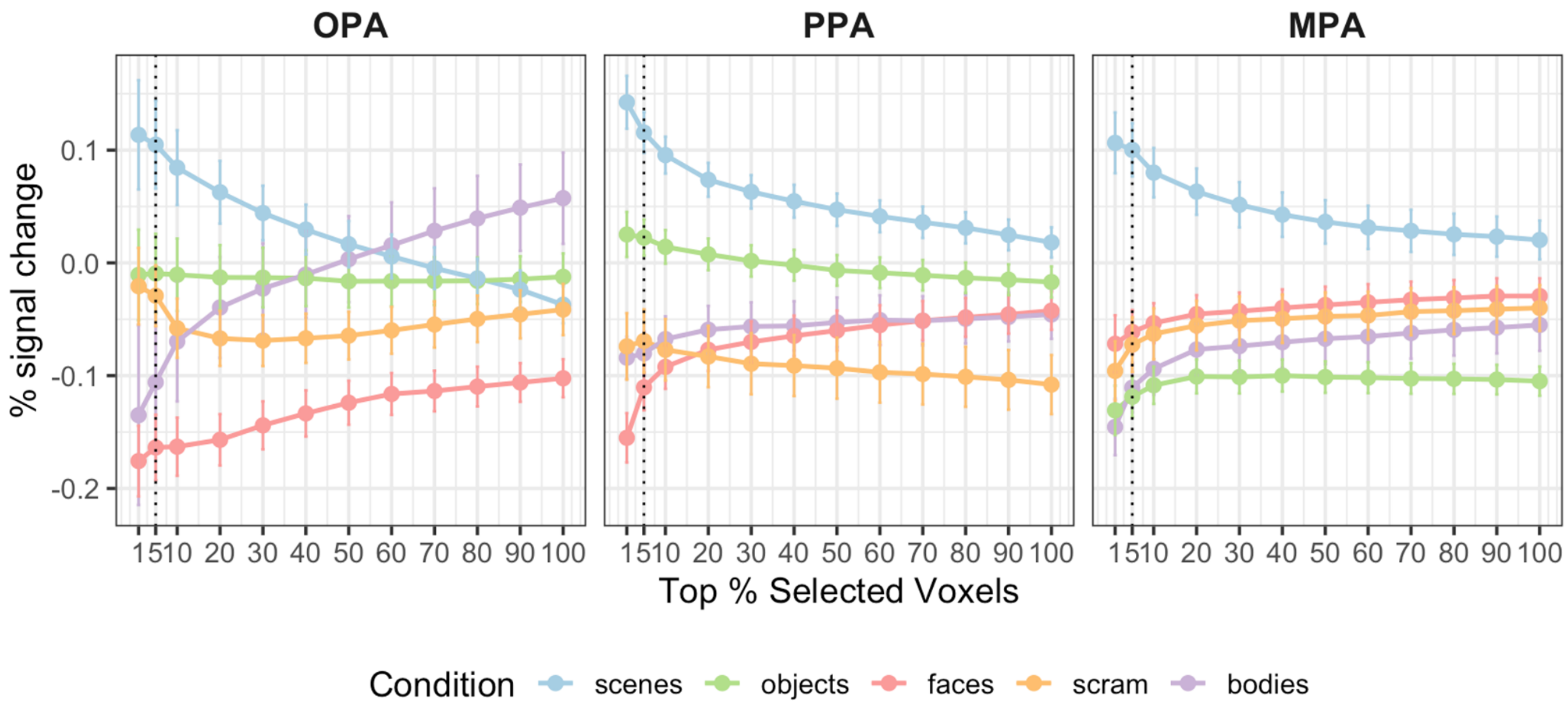

Supplemental Figure 4. How much of each anatomical parcel responds selectively to scenes? We conducted the same ssfROI analysis in each search space, varying the number of voxels selected from the top 1% of voxels to 100% of voxels. We then tested the response to scenes > all control conditions at each percentage. In OPA, we observe a selective response to scenes in held-out data when selecting anywhere from 1% to 40% of voxels, suggesting that scene-selective responses in infant OPA span about 11,712 mm3 of cortical territory. In PPA and MPA, the scene response is greater than control conditions even when 100% of voxels are included in the analysis, suggesting that scene responses cover at least 10,080 mm3 in PPA and at least 11840 mm3 in MPA. These results confirm that selective responses in infant ssfROIs are robust to analytic parameters for voxel selection.

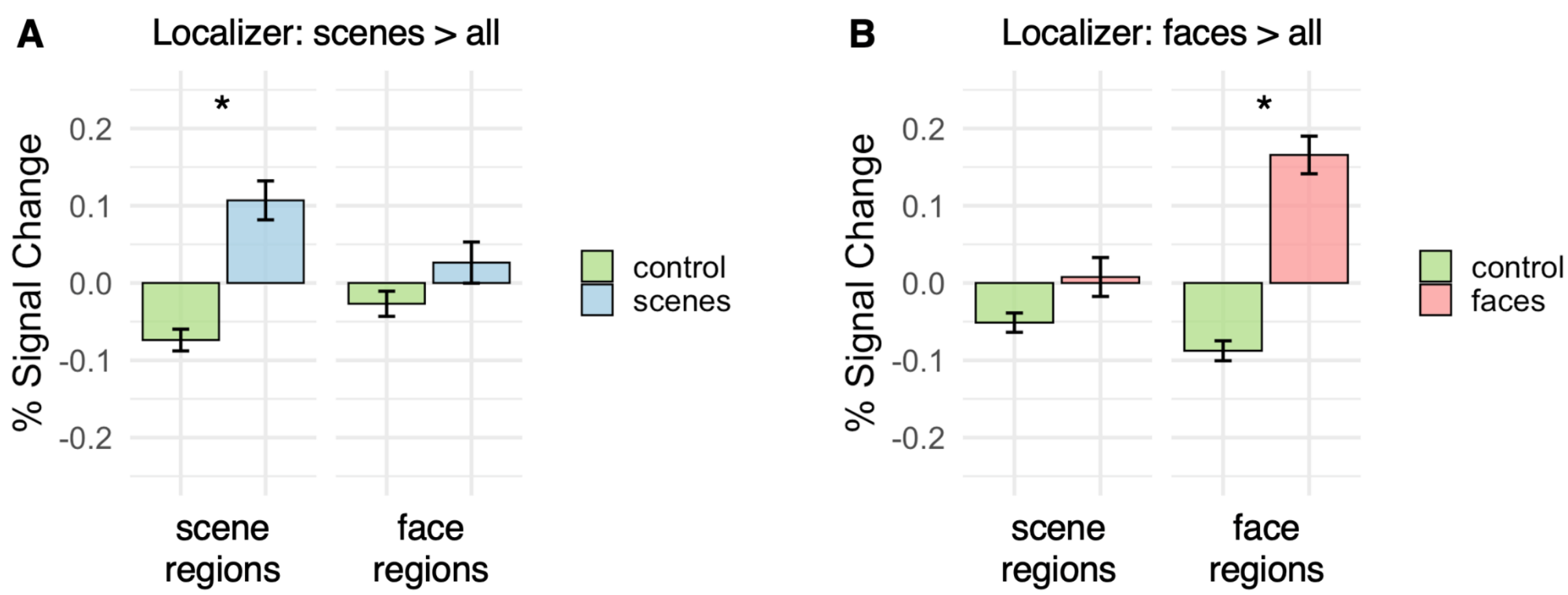

Supplemental Figure 5. *Scene and face selectivity are found in anatomically-expected areas.* To test whether the spatial organization of scene responses is similar to that in adults, we compared scene selectivity in scene regions to that in anatomical parcels for a network of face-selective regions that do not overlap with any known scene regions in adults, and which have previously been shown to contain face-selective regions in infancy (1, 6, 7). We also conducted the complementary ssfROI analysis testing face selectivity in both face and scene parcels. (A) Results of ssfROI analyses in anatomical parcels for scene and face regions, when top voxels are selected based on the contrast of scenes > all. (B) Results of ssfROI analyses in the same anatomical parcels for scene and face regions, when top voxels are now selected based on the contrast of faces > all. To maximize power and limit comparisons, we used the full dataset, and averaged results of ssfROI analyses across face and scene regions within each subject. Error bars indicate standard error of the mean.

Statistical analyses. Scene and face selectivity were assessed using a linear mixed effect model testing contrasts of scenes versus all other conditions (for tests of scene selectivity) or faces versus all other conditions (for face selectivity). Models also included factors for region (to account for overall differences in activation between face regions or between scene regions), experiment, age, and head motion (with a random intercept for subject). Consistent with the anatomical organization expected by adulthood, scene selectivity was detectable only in scene areas ($\beta = 0.05$, $t_{(395.22)} = 4.29$, $p < 0.001$), not face areas ($\beta = 0.01$, $t_{(538.89)} = 1.14$, $p = 0.13$), while face selectivity was detectable only in face areas ($\beta = 0.06$, $t_{(535.73)} = 6.51$, $p < 0.001$), not scene areas ($\beta = 0.02$, $t_{(393.46)} = 1.50$, $p = 0.07$). These results suggest that the spatial organization of scene (and face) selectivity in the infant cortex is similar to that in the adult cortex.

Lastly, we compared the strength of scene selectivity in scene areas to face selectivity in face areas. A linear mixed effect model (with factors for age, head motion, and experiment) found no significant difference in selectivity between face and scene regions ($\beta = 0.04$, $t_{(57.43)} = 1.52$, $p = 0.13$; two-tailed).

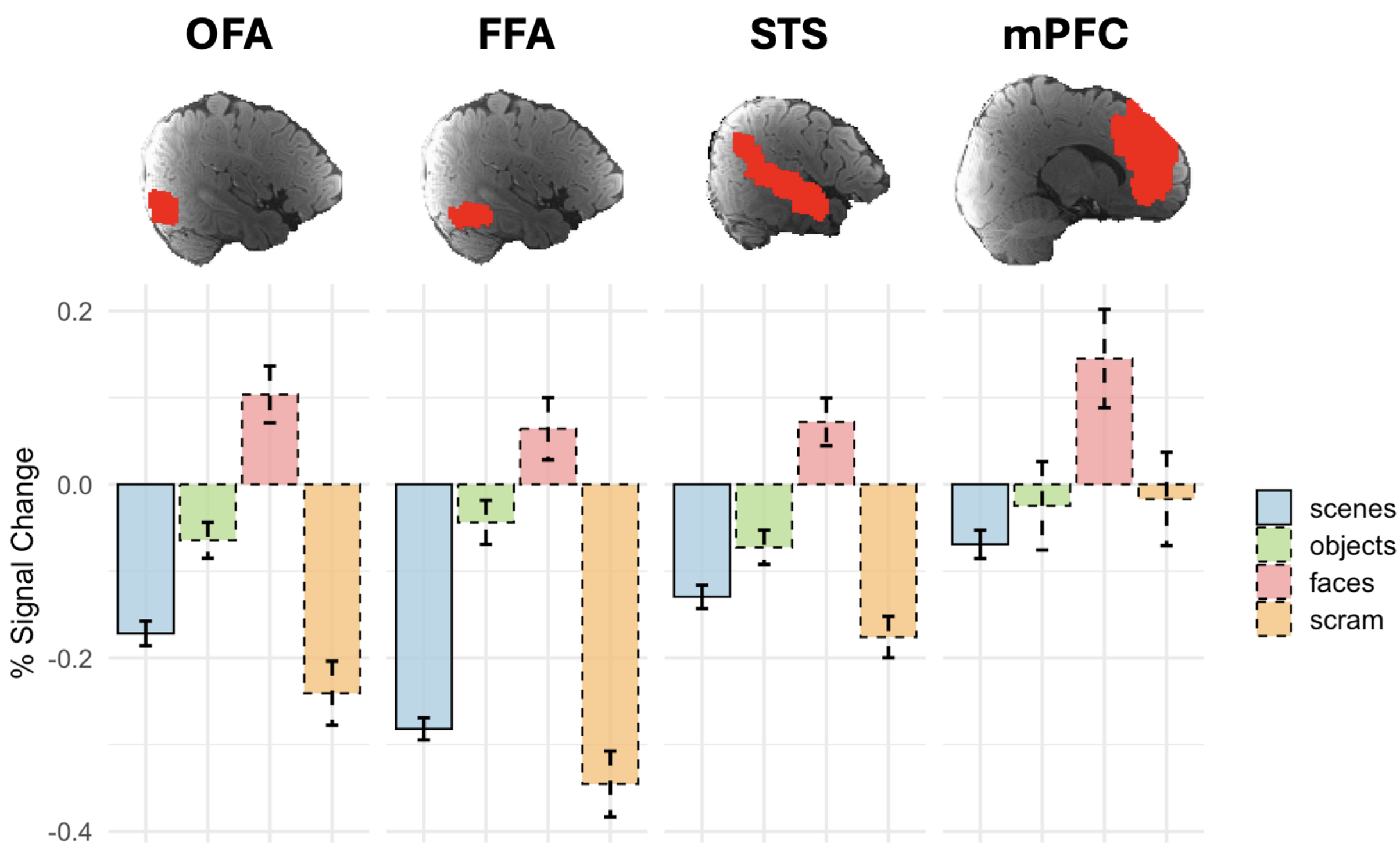

Supplemental Figure 6. *Independent replication of face-selectivity across the infant face network.* We conducted an exploratory ssfROI analysis testing for face selective activation using the preregistered dataset only (face selectivity in the infants from Kosakowski et al. were previously reported in refs 1 and 7 This analysis followed the same approach used to test scene selectivity, but now ranking voxels based on responses to faces > all in search spaces for four face-selective regions, including the occipital face area (OFA), fusiform face area (FFA), superior temporal sulcus (STS), and medial prefrontal cortex (mPFC). Replicating prior studies (1, 6, 7), a linear mixed effect model testing responses to faces versus the average of all other conditions revealed a stronger response to faces in each of the four regions (all β's > 0.04, all $t's_{(64.65)} > 1.66$, all $p's \leq 0.05$).

Supplemental Note 1

To define LO, we originally pre-registered plans to use the contrast of (objects and faces) > (scenes and scrambled scenes), but deviated from this plan after finding that this contrast tended to identify face-selective voxels. The contrast of objects > scenes also allowed us to use the same contrast for defining LO in the infants from Kosakowski et al. (1)

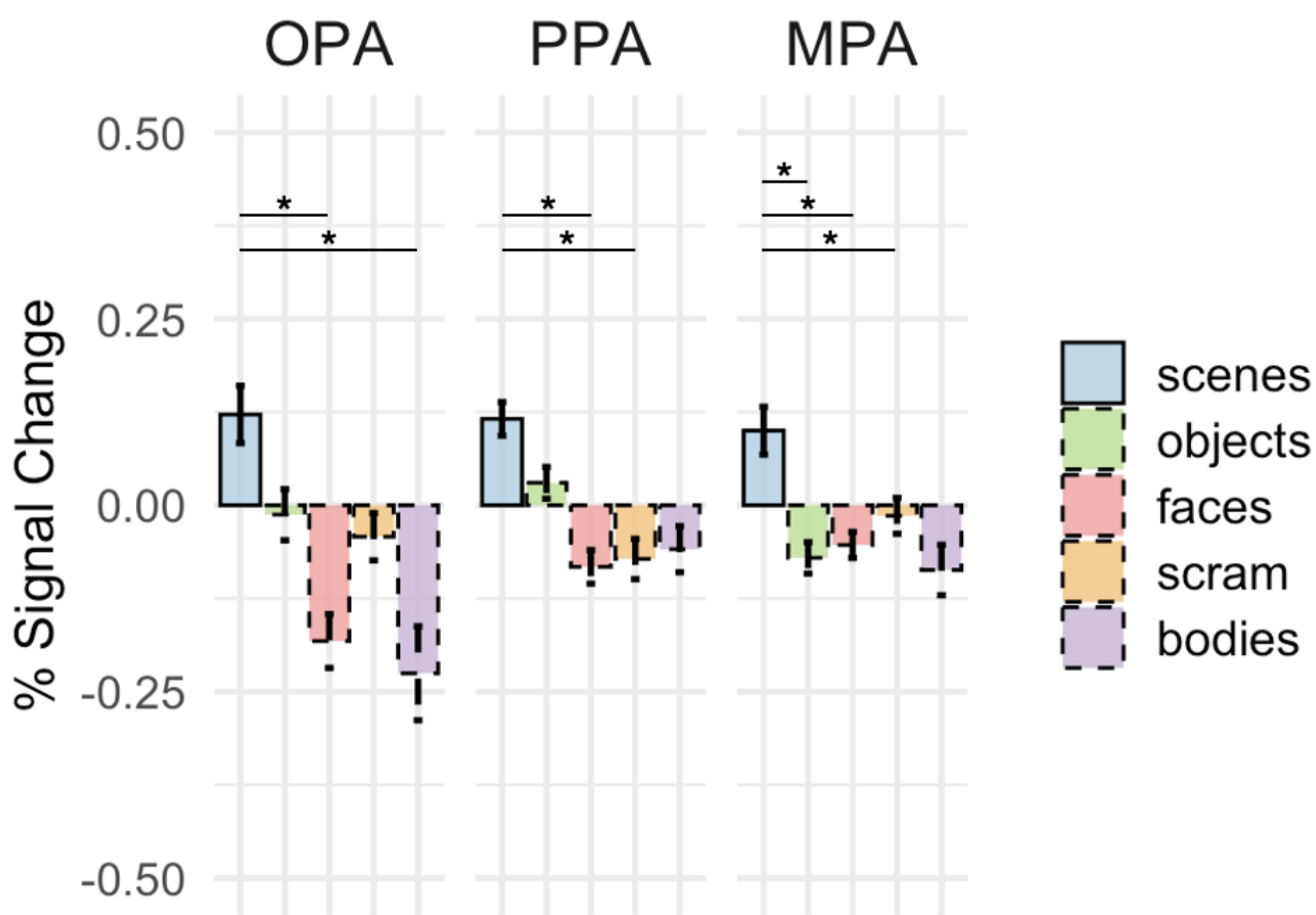

Supplemental Figure 7. *Responses to individual control conditions in pre-locomotor infants.* Due to the smaller number of infants in this sample, we analyzed data from the full sample of pre-locomotor infants only. Bar plots depict the mean response to scenes versus each individual control condition (objects, faces, and scrambled scenes). Dashed lines on control conditions reflect the fact that these conditions were sampled 33.3% as often as the scene condition (in the preregistered dataset only). Error bars indicate standard error of the mean. Lines and asterisks indicate control conditions where the response was significantly different from that to scenes in each region, based on results of a linear mixed effects model, as described in the main text.

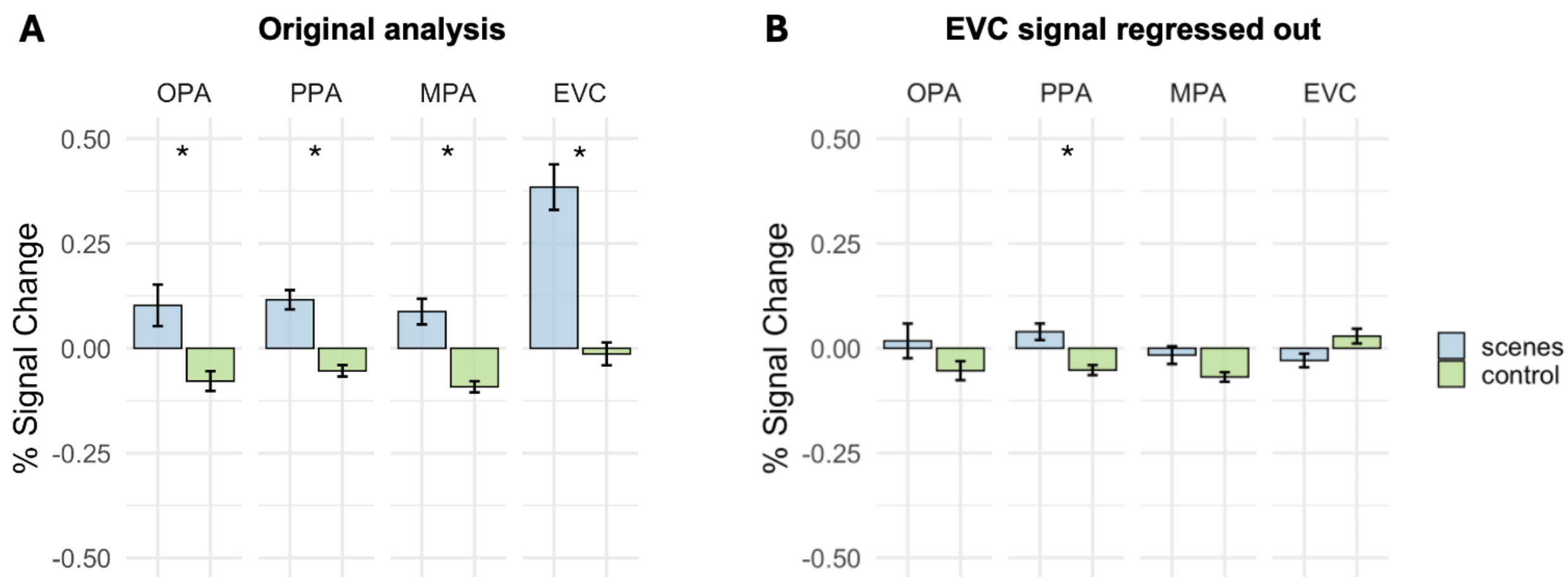

Supplemental Figure 8. *Scene-selectivity is partially, but not entirely, explained by responses in early-visual cortex (EVC).* (A) An exploratory ssfROI analysis in a search space for EVC revealed strong responses to scenes relative to control conditions. (B) To test whether this signal in EVC explains responses in infant scene cortex, we extracted the mean time course of activation across voxels from the scene-preferring EVC ssfROI, and reanalyzed each subrun including the EVC timecourse as a nuisance regressor in the GLM. Responses to scenes and control conditions were then re-extracted from the scene regions, as well as from the scene-preferring EVC (to confirm that the scene response had been eradicated in this region). Given that the scene-preferring EVC region is plausibly a key source of visual input to the infant scene regions, and given that timecourses between these nearby regions are likely to be correlated (reflecting both shared signal and shared noise), this analysis constitutes a high-bar for evidence of distinct representations in the infant scene regions vs. EVC. An exploratory omnibus ANOVA confirmed greater responses to scenes than control conditions could still be detected across the three scene regions ($\beta = 0.02$, $t_{(396.07)} = 1.87$, $p = 0.03$). When testing each scene region individually, greater responses to scenes than control conditions were found in PPA ($\beta = 0.02$, $t_{(107.55)} = 2.26$, $p = 0.01$), but did not reach significance in OPA ($\beta = 0.01$, $t_{(108.61)} = 0.86$, $p = 0.20$) or MPA ($\beta = 0.01$, $t_{(110.77)} = 1.23$, $p = 0.11$).

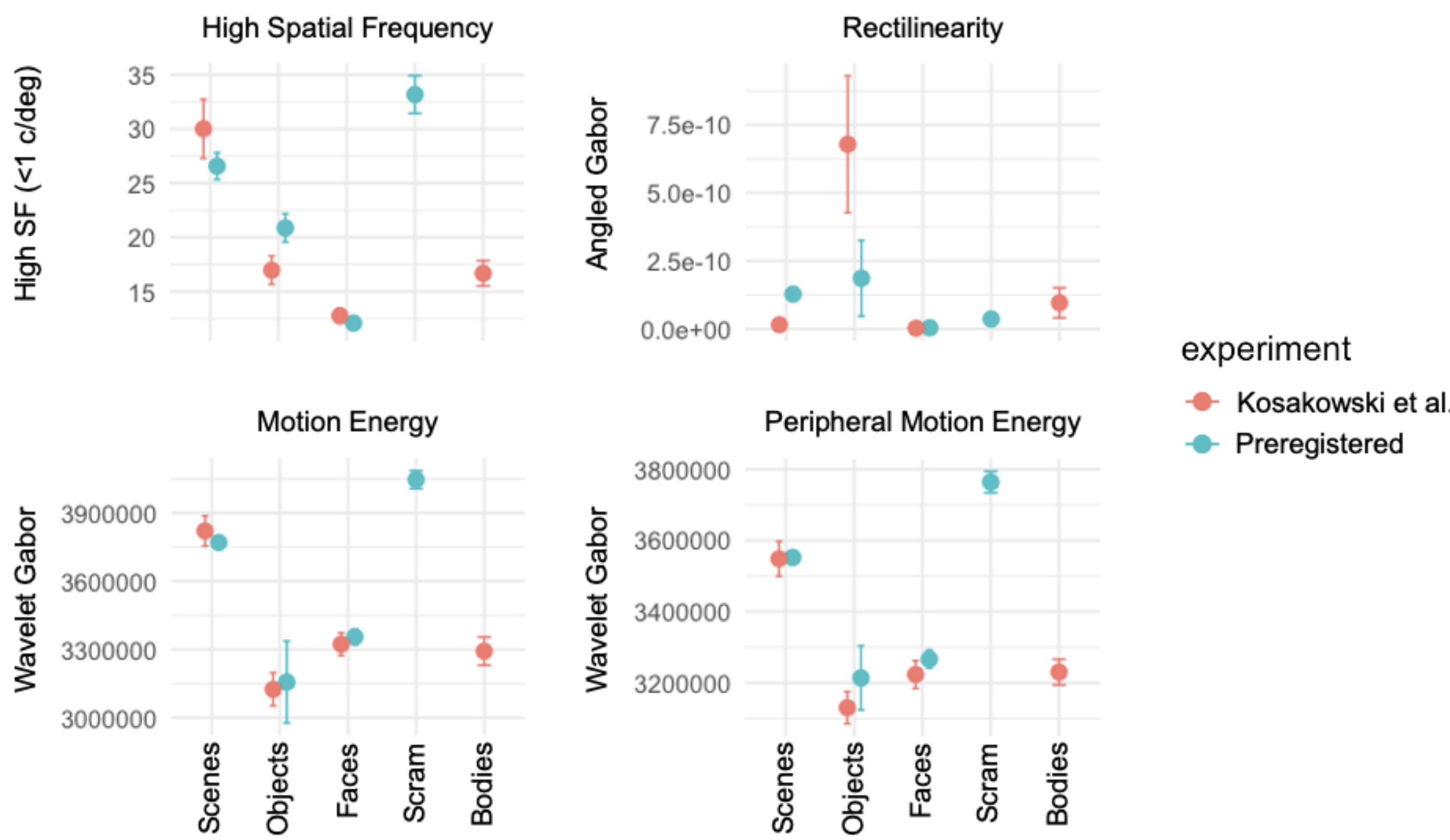

Supplemental Figure 9. *Average low-level visual feature content per stimulus category*. Rectilinearity, high spatial frequency, and motion energy (overall and in the periphery of the video frame) were estimated for each frame/stimulus and averaged within categories (see Methods). Each plot shows mean feature values for the stimuli in the preregistered dataset (blue dots) and in Kosakowski et al. (1) (red dots).

Effects of low-level features in individual scene regions

To further test whether responses in infant scene regions are better explained by scene selectivity vs. low-level features, we conducted exploratory analyses in individual scene regions. To do this, we used linear mixed effect models to test whether a categorical factor for scene selectivity explains significant variance in responses even when accounting for variance explained by low-level control features (each feature was tested individually against the categorical model). To maximize power for these analyses of individual regions, models were conducted on the full dataset.

For OPA, the effect of category was significant after accounting for rectilinearity (β = 0.08, $t_{(94.27)}$ = 2.07, p = 0.02), but did not reach significance after accounting for high spatial frequency, motion energy, or peripheral motion energy (all β's < 0.08, all t's$_{(94.27)}$ < 1.43, all p's ranged from 0.07-0.16). No low-level feature was significant after accounting for category (all β's < 0.03, all t's$_{(94.26)}$ < 1.39, all p's > 0.08). For PPA, the effect of category was significant after accounting for each of the four low-level features (all β's > 0.07, all t's$_{(93.61)}$ > 2.83, all p's < 0.003). Interestingly, there was a significant effect of rectilinearity after accounting for category (β = 0.02, $t_{(93.64)}$ = 2.03, p = 0.02), but no effects of any other low-level feature (all β's < 0.006, all t's$_{(93.64)}$ < 0.44, all p's > 0.16). For MPA, the effect of category was significant after accounting for each of the four low-level features (all β's > 0.09, all t's$_{(91.22)}$ > 3.12, all p's < 0.002). No low-level feature explained significant variance after accounting for category (all β's < -0.005, all t's$_{(91.27)}$ < -0.04, all p's > 0.39).

Representation of low-level features in non-scene stimuli

Studies of adults have found clearer evidence of low-level feature representation in scene regions using stimuli that lack space-defining features (e.g., 8, 9). Accordingly, we conducted an exploratory analysis testing whether low-level feature content could predict responses in scene regions for the non-scene control conditions only, which lack the clear definition of space found in the scene stimuli. However, none of the four low-level features predicted significant variance in infant scene region responses to the control categories, in either the preregistered dataset (all β's < 0.02, all $t's_{(167.89)} < 0.92$, all p's > 0.36) or the full dataset (all β's < 0.02, all $t's_{(247.27)} < 1.40$, all p's > 0.16).

Combinations of low-level features

It is possible that a combination of low-level visual features might be required to drive responses in infant scene regions, rather than any single feature. To test this idea, we created low-level feature combination models from all possible linear (the sum) and nonlinear (the product) combinations of the rectilinearity (R), high spatial frequency (H), and motion energy (M) features (i.e., R+H, R+M, H+M, R+H+M, R*H, R*M, H*M, and R*H*M). Each raw feature vector was rescaled from 0 to 1 prior to adding or multiplying, to place features on the same scale and avoid sign-flipping issues caused by negative values. We then scaled the low-level feature combination models, and compared each model individually to the categorical model using the same linear mixed effect model approach described above. Once again, we found (using the full dataset) that the categorical model remained significant after accounting for each of the eight possible combined low-level feature models (all β's > 0.07, all t's > 2.76, all p's < 0.006). No linear or non-linear combined low-level feature model explained significant variance after accounting for the category model (all β's < 0.02, all t's < 1.45, all p's > 0.14).

Age-related change for individual conditions

Here we tested for age-related change in the response to each individual condition (i.e., % signal change to scenes, objects, faces, scrambled scenes, or bodies), controlling for motion, total volumes, and variance explained by the GLM. For both the preregistered dataset and full datasets, we found no effect of age (either positive or negative; beta and t values reported as absolute values) for any condition in any region (all $|\beta|$'s < 0.16, all $|t|$'s < 2.18, all p's > 0.05), with the exceptions of OPA – which showed an increasing response with age to scrambled scenes ($\beta = 0.09$, $t = 2.95$, $p = 0.005$) and bodies ($\beta = 0.18$, $t = 3.33$, $p = 0.003$) – and PPA, which showed a decreasing response with age to bodies ($\beta = -0.05$, $t = 3.03$, $p = 0.005$).

Age-related change after controlling for EVC

We also tested for age-related change in scene responses after controlling for EVC (i.e., using the responses to scenes versus control conditions estimated after regressing out the timecourse from scene-preferring EVC voxels). Specifically, using the responses to scenes versus control conditions estimated after regressing out the timecourse from scene-preferring EVC voxels, we fit exploratory linear models with regressors for "scenes_vs_control", age, and their interaction (as well as additional factors for experiment, motion, total volumes, and variance explained by the GLM). Analyses were conducted using the full dataset only to maximize power. These analyses failed to find evidence of an age by scene selectivity interaction in each of the three regions (all $|\beta|$'s < 0.02, all $|t|$'s < 1.20, all p's > 0.23). We also failed to find an age by scene selectivity interaction in a model combining data from all three regions (with an additional factor for region) ($\beta = 0.001$, $t = 0.13$, $p = 0.90$).

Additional tests of scene selectivity in young infants

Here we report tests of scene selectivity in the younger (median split) age group, using the preregistered dataset only. This younger group included a small sample of just 11 sessions from 10 infants (mean age = 3.60 months, range = 2.07-5.33 months), and should therefore be interpreted with caution. As with the younger group in the full sample, an exploratory omnibus analysis (using data from all three scene regions) in this younger group from the preregistered sample revealed a significantly greater response to scenes than control conditions (β = 0.03, $t_{(117.42)}$ = 1.87, p = 0.03).

We also analyzed responses to individual control conditions in the younger age group, including data from the full dataset only to maximize power. Results for each ssfROI and each contrast of scenes versus a control condition are summarized below in Supplemental Table 2.

Supplemental Table 2

Full dataset

Region	Control contrast	beta	df	t value	p value	p<0.05
OPA	object	-0.04	58.56	-0.69	0.24	
OPA	face	-0.13	58.56	-2.32	0.01	*
OPA	scram	-0.08	58.56	-1.07	0.15	
OPA	bodies	-0.19	58.56	-2.61	0.006	*

Region	Control contrast	beta	df	t value	p value	p<0.05
PPA	object	-0.04	59.52	-1.20	0.12	
PPA	face	-0.07	59.52	-2.14	0.02	*
PPA	scram	-0.11	59.52	-2.49	0.008	*
PPA	bodies	-0.06	59.52	-1.33	0.095	

Region	Control contrast	beta	df	t value	p value	p<0.05
MPA	object	-0.07	59.51	-1.92	0.03	*
MPA	face	-0.06	59.51	-1.52	0.07	
MPA	scram	-0.07	59.51	-1.51	0.07	
MPA	bodies	-0.05	59.51	-1.08	0.14	

Supplemental Note 2

Notably, our claim that basic scene selectivity emerges across the scene network in infancy does not imply that scene selectivity is fully developed, adult-like, or even child-like. While we find greater responses to scenes than control conditions in anatomically similar regions to older groups – the basic criterion for scene selectivity – the magnitude of responses and effect size estimates are much weaker than those previously observed in older children and adults (e.g., 10-15). Direct comparison of the current data from awake infants to that from older age groups is a significant challenge, as many factors likely differ between infants and older age groups. For example, infants have smaller, slower, and more variable hemodynamic responses (16); greater head motion (despite the strict thresholds we applied); weaker attention to stimuli; lower temporal signal to noise; and poorer registration – all of which will reduce response estimates relative to older groups. We hope our results help pave the way for future work that might overcome these challenges to test developmental change across years of child development.

Study	Subject ID	Age (Months)	Sex	Total Volumes
Preregistered	inf01	8.84	m	212
Preregistered	inf02	9.11	f	255
Preregistered	inf03	2.73	f	199
Preregistered	inf04	5.92	f	345
Preregistered	inf05	6.51	m	633
Preregistered	inf06	7.07	f	345
Preregistered	inf07	2.56	f	230
Preregistered	inf08	5.33	f	389
Preregistered	inf08	7.36	f	738
Preregistered	inf09	4.18	f	436
Preregistered	inf10	6.64	f	429
Preregistered	inf10	8.78	f	267
Preregistered	inf11	5.69	m	769
Preregistered	inf11	7.89	m	470
Preregistered	inf12	2.99	f	408
Preregistered	inf13	2.07	m	651
Preregistered	inf14	3.06	m	890
Preregistered	inf15	4.83	f	414
Preregistered	inf16	2.53	f	608
Preregistered	inf16	4.44	f	792
Preregistered	inf17	4.93	f	1542
Kosakowski	inf01	5.18	m	772
Kosakowski	inf02	4.66	m	1182
Kosakowski	inf03	9.41	f	760
Kosakowski	inf04	4.56	m	324
Kosakowski	inf05	6.00	f	541
Kosakowski	inf06	5.57	m	311
Kosakowski	inf07	4.00	f	449
Kosakowski	inf08	4.43	f	317
Kosakowski	inf09	6.72	m	207
Kosakowski	inf10	4.30	m	672
Kosakowski	inf11	6.43	m	332
Kosakowski	inf12	2.98	f	463
Kosakowski	inf13	3.77	f	651
Kosakowski	inf14	4.43	f	543
Kosakowski	inf15	3.21	m	485

Supplemental Table 3. *Subject and session information.* Each row is a session, where a session is the combined usable data across visits conducted within a 30 day window. Columns from left to right indicate: (1) the study in which the data were collected; (2) the Subject ID; (3) the age of the infant in months at for the first visit of that session; (4) sex reported; (5) the total volumes collected across visits, summed across usable subruns.

References

1. Kosakowski, H. L., Cohen, M. A., Takahashi, A., Keil, B., Kanwisher, N., & Saxe, R. (2022). Selective responses to faces, scenes, and bodies in the ventral visual pathway of infants. *Current Biology*, *32*(2), 265-274.
2. Chen, E. M.*, Kamps, F. S.*, & Saxe, R. R. (2024). An open dataset of functional MRI responses to egocentric navigation through natural scenes. *7th Annual Conference on Cognitive Computational Neuroscience*. Cambridge, Massachusetts, USA.
3. Paruthi, S., Brooks, L. J., D'Ambrosio, C., Hall, W. A., Kotagal, S., Lloyd, R. M., ... & Wise, M. S. (2016). Recommended amount of sleep for pediatric populations: a consensus statement of the American Academy of Sleep Medicine. *Journal of clinical sleep medicine*, *12*(6), 785-786.
4. Daw, N. W., & Daw, N. W. (2006). *Visual development* (Vol. 14). New York: Springer.
5. Atkinson, Janette, 'Newborn vision', *The Developing Visual Brain*, Oxford Psychology Series (Oxford, 2002; online edn, Oxford Academic, 1 Jan. 2008), https://doi.org/10.1093/acprof:oso/9780198525998.003.0004.
6. Deen, B., Richardson, H., Dilks, D. D., Takahashi, A., Keil, B., Wald, L. L., ... & Saxe, R. (2017). Organization of high-level visual cortex in human infants. *Nature communications*, *8*(1), 13995.
7. Kosakowski, H. L., Cohen, M. A., Herrera, L., Nichoson, I., Kanwisher, N., & Saxe, R. (2024). Cortical face-selective responses emerge early in human infancy. *eneuro*, *11*(7).
8. Bryan, P. B., Julian, J. B., & Epstein, R. A. (2016). Rectilinear edge selectivity is insufficient to explain the category selectivity of the parahippocampal place area. *Frontiers in human neuroscience*, *10*, 137.
9. Nasr, S., Echavarria, C. E., & Tootell, R. B. (2014). Thinking outside the box: rectilinear shapes selectively activate scene-selective cortex. *Journal of Neuroscience*, *34*(20), 6721-6735.
10. Golarai, G., Ghahremani, D. G., Whitfield-Gabrieli, S., Reiss, A., Eberhardt, J. L., Gabrieli, J. D., & Grill-Spector, K. (2007). Differential development of high-level visual cortex correlates with category-specific recognition memory. *Nature neuroscience*, *10*(4), 512-522.
11. Jung, Y., Hsu, D., & Dilks, D. D. (2024). “Walking selectivity” in the occipital place area in 8-year-olds, not 5-year-olds. *Cerebral Cortex*, *34*(3), bhae101.
12. Kamps, F. S., Pincus, J. E., Radwan, S. F., Wahab, S., & Dilks, D. D. (2020). Late development of navigationally relevant motion processing in the occipital place area. *Current Biology*, *30*(3), 544-550.
13. Meissner, T. W., Nordt, M., & Weigelt, S. (2019). Prolonged functional development of the parahippocampal place area and occipital place area. *Neuroimage*, *191*, 104-115.
14. Scherf, K. S., Behrmann, M., Humphreys, K., & Luna, B. (2007). Visual category-selectivity for faces, places and objects emerges along different developmental trajectories. *Developmental science*, *10*(4), F15-F30.
15. Scherf, K. S., Luna, B., Avidan, G., & Behrmann, M. (2011). “What” precedes “which”: developmental neural tuning in face-and place-related cortex. *Cerebral cortex*, *21*(9), 1963-1980.

16. Arichi, T., Fagiolo, G., Varela, M., Melendez-Calderon, A., Allievi, A., Merchant, N., ... & Edwards, A. D. (2012). Development of BOLD signal hemodynamic responses in the human brain. *Neuroimage*, *63*(2), 663-673.
